# Heme delivery into soluble guanylyl cyclase requires a heme redox change and is regulated by NO and Hsp90 by distinct mechanisms

**DOI:** 10.1016/j.jbc.2025.108315

**Published:** 2025-02-13

**Authors:** Yue Dai, Dennis J. Stuehr

**Affiliations:** Department of Inflammation and Immunity, Cleveland Clinic Lerner College of Medicine of Case Western Reserve University School of Medicine, Cleveland, Ohio, USA

**Keywords:** nitric oxide, guanylyl cyclase, GAPDH, heat shock protein 90, heme, heme transfer

## Abstract

Nitric oxide (NO) signaling often relies on it activating cGMP production by the heterodimeric enzyme soluble guanylyl cyclase (sGC). To mature to function, an sGCβ subunit must first incorporate heme and then form a heterodimer with a partner α subunit. Our previous studies in cells showed that glyceraldehyde 3-phosphate dehydrogenase (GAPDH) supplies heme to the apo-sGCβ subunit, which is complexed with the cell chaperone Hsp90. Through its ATP hydrolysis, Hsp90 then promotes heme insertion into apo-sGCβ and consequent formation of a functional heterodimer. NO at physiologic levels somehow stimulates cell heme allocation into apo-sGCβ by this process. To gain insight, we utilized purified apo-sGCβ and GAPDH reporter proteins whose heme contents can be followed by fluorescence and determined the impact of Hsp90 and NO on heme transfer between them. Results show that heme transfer out of GAPDH and into apo-sGCβ is tightly coupled in all circumstances and is limited by the ability of the apo-sGCβ to incorporate the heme, which in turn relies on a ferric to ferrous heme transition taking place inside the sGCβ. Hsp90 can influence the heme transfer kinetics in a negative or positive manner through its conformational effects on apo-sGCβ, while NO speeds heme transfer by binding to the heme iron and thus speeding heme dissociation from GAPDH. Our findings provide new mechanistic understanding of sGC maturation and how Hsp90 and NO combine to dynamically regulate heme incorporation for sGC heterodimer formation and consequent cGMP production in biological settings.

Impacts of nitric oxide (NO) that involve it activating cGMP production by the enzyme soluble guanylyl cyclase (sGC) (1, 2) include vasorelaxation (3), peristalsis (4), immune surveillance (5), reproductive processes (6), bronchodilation (7), and neural functions (8). NO activates by binding to the iron atom of a heme cofactor that is bound within the sGCβ subunit of a sGCα/β heterodimer, which in turn causes the protein structural changes that activate its catalysis (9). The essential role of the heme makes it important to understand how it is delivered and becomes inserted into the sGCβ subunit during the enzyme's functional maturation in cells (10, 11). Our recent studies on sGCα/β maturation have employed an sGCβ reporter construct originally developed by Hoffmann *et al.* (12) that utilizes fluorescein arsenical hairpin binder (FlAsH) technology developed by Tsien et al. (13). The sGCβ reporter construct has a tetra-Cys–containing sequence (TC) inserted near its heme-binding site, thus creating TC-sGCβ (14). When the TC sequence is labeled with the arsenical dye FlAsH (15), the TC-sGCβ protein becomes fluorescent and its emission intensity is inversely proportional to its bound heme content. This allows one to track changes in the TC-sGCβ heme content in real time both for the purified protein and when it is expressed in living cells (12, 14). Overall, we have learned that following its translation in mammalian cells, the sGCβ subunit is heme-free (apo-sGCβ) and in complex with heat shock protein 90 (Hsp90) (11, 14), a cell chaperone that generally acts through conformational mechanisms to enable the end folding of numerous protein clients and/or their uptake of small ligands (16). Glyceraldehyde-3-phosphate dehydrogenase (GAPDH) transports intracellular heme and provides it to the apo-sGCβ–Hsp90 complex (17, 18), and the heme insertion is driven by the bound Hsp90 *via* its ATP hydrolysis activity (10, 11, 14). Hsp90 then dissociates from the heme-replete sGCβ subunit, and this allows combination with a sGCα partner subunit to form the mature sGCα/β heterodimer that can respond to NO and generate cGMP for signaling (10, 14). More recently, we have employed the same FlAsH/TC approach to create a TC-GAPDH reporter construct that allows monitoring of its heme binding and release in living cells or in purified form (19). Thus, the FlAsH/TC technology can allow one to follow either heme release by the donor protein (GAPDH) or heme acquisition by the acceptor protein (apo-sGCβ).

Several studies suggest that heme is naturally kept in deficit in cells and tissues and this causes many heme proteins to accumulate in their heme-free forms (20, 21). This includes sGCβ, which has been estimated to exist 30 to 80% in its heme-free form in mammalian cells and tissues (7, 21, 22). In this context, we have found that NO can regulate in an additional way by causing cells to redistribute their heme into their apo-sGCβ subpopulation (23). Indeed, using TC-sGCβ, we found that exposing cells to physiologic levels of NO immediately triggered them to start redistributing heme into apo-TC-sGCβ, which in turn led to a several fold increase in the cell's level of functionally active sGCα/β heterodimer (23). The NO-driven redistribution of cellular heme into apo-sGCβ depended on cell GAPDH and Hsp90 (23), suggesting it proceeds by the same mechanism as does cell heme provision during normal sGCα/β maturation. Although this stands to be an important new way that NO can regulate cGMP signaling in biology, fundamental gaps remain in our understanding of how GAPDH, Hsp90, and NO enable heme delivery and insertion into apo-sGCβ.

To help fill these gaps, we have developed an *in vitro* system to study heme transfer that utilizes purified versions of the TC-GAPDH, Hsp90, and TC-sGCβ proteins. In this way, we could observe in real time the heme transfer from a FlAsH-labeled TC-GAPDH–heme complex to apo-sGCβ and, reciprocally, observe the heme insertion into a FlAsH-labeled apo-TC-sGCβ from a preformed GAPDH–heme complex. Our findings reveal that heme transfer between GAPDH and apo-sGCβ is tightly coupled, depends on direct protein–protein interaction, involves the ferric heme undergoing a redox change, and is regulated by Hsp90 and NO through two different mechanisms.

## Results

All of our studies utilized a bacterially expressed truncated form of rat sGCβ that contains amino acids 1 to 385 out of 619 total (designated sGCβ for simplicity) (17, 24). The truncated protein contains the N-terminal heme-binding domain, the downstream per-arnt-sim (PAS) domain, and a portion of a linker that in the full-length sGCβ connects the PAS domain to the coiled coil domain and downstream catalytic domain. This truncated protein construct has been used in place of the full-length sGCβ in numerous studies (14, 25, 26) due to a general inability to overexpress the full-length subunit in bacteria. Importantly, the truncated sGCβ protein we used here contains amino acid sequence elements in the PAS and linker regions that enable it and the full-length sGCβ subunit to bind and respond to Hsp90 (14, 27).

### GAPDH transfers heme to apo-sGC**β** in a concerted and efficient manner

**Figure 1:**
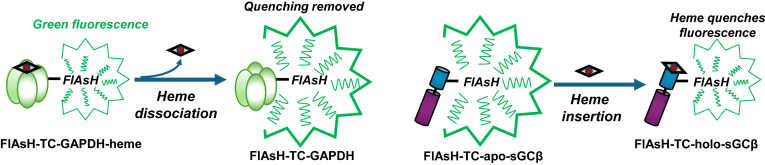
**Monitoring heme binding in TC-sGCβ and TC-GAPDH.** The fluorescence intensity of either FlAsH-labeled protein is inversely proportional to its heme content, allowing changes in their heme contents to be followed *versus* time.

We first investigated heme transfer in a simple two-component reaction in which GAPDH loaded with a physiologic level of ferric or ferrous heme (approximately one heme per GAPDH tetramer) (19, 28) was mixed with a FlAsH-labeled TC-apo-sGCβ and in reciprocal reactions where a FlAsH-labeled TC-GAPDH containing ferric or ferrous heme was mixed with apo-sGCβ. This allowed us to separately follow the rate of heme incorporation into FlAsH-apo-TC-sGCβ and the rate of heme loss from FlAsH-TC-GAPDH in the reciprocal reactions by following the decrease or increase in the FlAsH fluorescence emission intensity, respectively (Fig. 1) (14, 19). All reactions were run at 25 °C.

**Figure 2:**
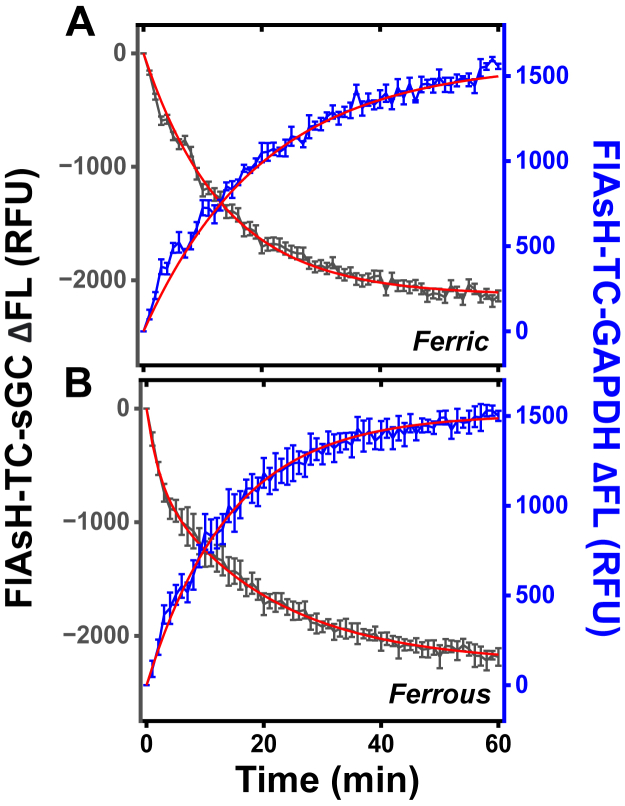
**FlAsH fluorescence traces indicating the kinetics of ferric or ferrous heme transfer from GAPDH and into apo-sGCβ.** Reactions contained 1 μM each of the FlAsH-labeled and unlabeled partner proteins and were initiated by adding the unlabeled partner protein. Representative traces are the mean ± SD of three replicate reactions and show the gain or loss in fluorescence intensity *versus* time that indicate the loss of ferric heme (*A*) or ferrous heme (*B*) from FlAsH-TC-GAPDH or their incorporation into FlAsH-TC-apo-sGCβ during the reactions, respectively. *Red* lines are the fitted curves.

Figure 2*A* contains representative averaged fluorescence traces recorded in reactions where 1 μM of GAPDH tetramer containing approximately one ferric heme was mixed with 1 μM FlAsH-TC-apo-sGCβ or where 1 μM apo-sGCβ was mixed with 1 μM FlAsH-TC-GAPDH ferric heme complex. We also performed similar reactions under anaerobic conditions using premade complexes of GAPDH or FlAsH-TC-GAPDH containing ferrous heme (Fig. 1*B*). In both cases (for ferric or ferrous heme), we observed a time-dependent and reciprocal change in the fluorescence emission intensities of the FlAsH-apo-TC-sGCβ and FlAsH-TC-GAPDH that reached completion near 60 min after initiating the reactions. Fitting these traces to a single exponential function gave observed rates for ferric or ferrous heme loss from FlAsH-TC-GAPDH of 0.06 ± 0.01 and 0.07 ± 0.02 min^−1^, respectively, and for ferric or ferrous heme gain into FlAsH-TC-apo-sGCβ of 0.05 ± 0.02 and 0.05 ± 0.01 min^−1^, respectively. Because there was no large difference between the ferric or ferrous heme transfer kinetics in our reaction system, we performed most of our subsequent studies using GAPDH or FlAsH-TC-GAPDH containing ferric heme.

Based on the known extent of fluorescence quenching that occurs when one ferric heme binds into a FlAsH-TC-GAPDH tetramer (22%) (19) and when one ferric heme binds into a FlAsH-apo-TC-sGCβ (64%, Fig. S1), we could estimate from the percentage of fluorescence loss or gain that occurred in the experimental traces of Figure 2, *A* and *B* that 84% of the heme bound in FlAsH-TC-GAPDH had left the protein over the course of the reaction, and this corresponded to a 83% heme gain into FlAsH-TC-apo-sGCβ in the reciprocal experiment. Also, we found that a close relationship existed between the rates of ferric heme loss from GAPDH and heme gain into apo-sGCβ across a range of reactions that contained a fixed concentration of GAPDH heme tetramer plus a varied concentration of FlAsH-TC-apo-sGCβ acceptor or alternatively contained a fixed concentration of apo-sGCβ acceptor and a varied concentration of FlAsH-TC-GAPDH heme tetramer (Fig. S2, *A*–*C*). To investigate the kinetics of GAPDH and apo-sGCβ complex formation in our reactions, we monitored the change in residual fluorescence polarization *versus* time of either FlAsH-tagged protein after mixing it in a 1:1 ratio with its nonlabeled partner to give 1 μM final concentrations. We observed a gain in the residual polarization within the mixing dead time (<3 min) in both circumstances that increased no further with time (Fig. S3, *A* and *B*). This indicated that protein complex formation between GAPDH and apo-sGCβ was relatively quick and should not limit the rates of heme transfer that we observed in the reactions. To investigate how important is the direct protein interaction between the GAPDH–heme complex and apo-sGCβ for heme transfer, we compared the rates of heme delivery from a GAPDH–heme complex when it was mixed with FlAsH-TC-apo-sGCβ alone or was mixed with a preformed complex consisting of GAPDH already bound on FlAsH-TC-apo-sGCβ, reasoning that if a direct protein interaction enabled the heme transfer, then having GAPDH pre-bound to the FlAsH-TC-apo-sGCβ should antagonize heme delivery from the GAPDH–heme complex. Fig. S4 shows that the heme transfer from the GAPDH–heme complex to FlAsH-TC-apo-sGCβ alone fit to a single exponential and completed within 30 min. In contrast, heme transfer to the preformed GAPDH–FlAsH-TC-apo-sGCβ complex exhibited biphasic kinetics with the second phase dominating and being five times slower, resulting in incomplete heme transfer after 60 min. This indicated that the heme transfer was retarded when a direct interaction of the GAPDH–heme complex with apo-sGCβ was antagonized. Together, the results indicate that heme transfer from GAPDH into apo-sGCβ proceeded in a direct, concerted, and efficient manner under our reaction conditions.

### Hsp90 speeds heme transfer from GAPDH to apo-sGC**β** in an ATP-dependent manner

**Figure 3:**
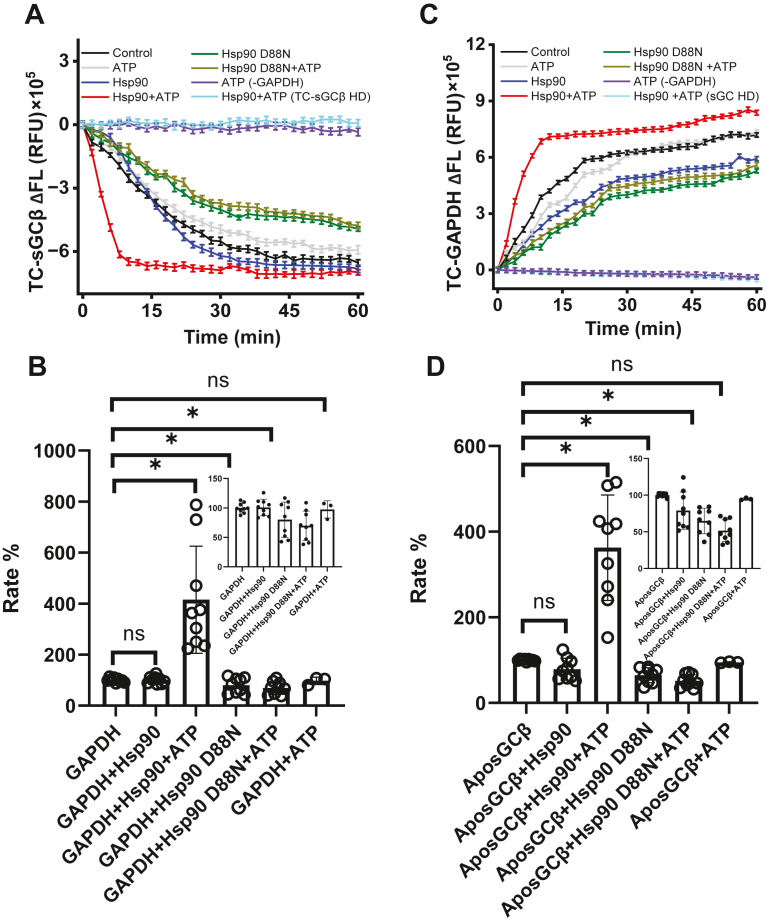
**The impact of Hsp90 and its ATPase activity on heme transfer from GAPDH to apo-sGCβ.** Reactions contained 1 μM each of unlabeled or FlAsH-labeled versions of the TC-GAPDH-heme complex or TC-apo-sGCβ either alone or in complex with Hsp90. Reactions were initiated by adding the unlabeled partner protein. In some cases, ATP was present or a variant of Hsp90 (D88N) or TC-apo-sGCβ (HD) were used as indicated. (*A* and *C*) contain representative fluorescence traces (mean ± SD of three replicates) that indicate (*A*) the kinetics and extent of heme gain into the indicated FlAsH-labeled TC-apo-sGCβ proteins or (*C*) heme loss from FlAsH-TC-GAPDH in companion reactions. The relative rates of heme gain and heme loss under the various reaction conditions are compared in (*B* and *D*), respectively. Data points in (*B* and *D*) are derived from three independent trials, and the changes observed in each trial were normalized by assigning a value of 100 to the mean rate (from n = 3 replicates) obtained for heme transfer in the simple two-component reaction run in each trial. Inserts show the data using a smaller y-axis scale. ∗*p* ≤ 0.05, significantly different as indicated. HD, heme binding deficient; ns, nonsignificant.

In cells, apo-sGCβ is found in complex with Hsp90, which drives the heme insertion *via* its ATPase activity (29). To study how Hsp90 may impact heme transfer from the GAPDH–heme complex to apo-sGCβ in our defined reaction system, we first determined the concentration of Hsp90 that would allow full complex formation with the apo-sGCβ (Fig. S5) and also confirmed that binding Hsp90 to FlAsH-TC-apo-sGCβ did not change its intrinsic heme-binding affinity or its degree of fluorescence quenching upon binding 1 mol/mol of heme (Fig. S1). Figure 3*A* contains representative averaged fluorescence traces recorded after a GAPDH–heme complex was mixed under the indicated reaction conditions with either FlAsH-TC-apo-sGCβ alone or with FlAsH-TC-apo-sGCβ that had been preincubated with 5 μM Hsp90 to allow for their complex formation. Fluorescence traces from the trials and their fitted rates are shown in Fig. S6 and the resulting mean rates ± SD and calculated extents of heme transfer are listed in Table S1. Figure 3*B* plots the heme transfer rates obtained for each reaction condition as percentages relative to the mean rate obtained in the simple two-component reaction (set to 100%) that was run in each trial. Hsp90 complexation with FlAsH-TC-apo-sGCβ had no impact on its rate of heme incorporation, but when ATP was included in the reaction, the rate of heme incorporation increased by two- to seven-fold (Fig. 3, *A* and *B*). The degree of rate enhancement caused by Hsp90 and ATP was independent of the concentration of the GAPDH–heme complex added to the reaction (Fig. S7). No heme transfer occurred (as judged by no change in fluorescence intensity *versus* time) when a variant of sGCβ that is unable to bind heme (17) (FlAsH-apo-TC-sGC HD) was used in the reaction (Fig. 3, *A* and *B*). When we utilized a Hsp90 variant (D88N) that cannot hydrolyze ATP but otherwise displayed normal binding affinity toward FlAsH-TC-apo-sGCβ (Fig. S5), the rate of heme incorporation into FlAsH-TC-apo-sGCβ was reduced by 25% and addition of ATP no longer caused any rate increase (Fig. 3, *A* and *B*). In the absence of Hsp90, adding ATP alone had no effect (Fig. 3, *A* and *B*). Thus, when Hsp90 was bound to FlAsH-TC-apo-sGCβ, it could only increase the rate of heme transfer from GAPDH when it possessed a functional ATPase activity and ATP was present.

In parallel reactions, we investigated the impact of Hsp90 and ATP on the rate of heme loss from FlAsH-TC-GAPDH. Reactions were initiated by mixing apo-sGCβ or the apo-sGCβ–Hsp90 complex with a FlAsH-TC-GAPDH heme complex under the various conditions described above. Representative fluorescence traces showing the kinetics of heme loss from FlAsH-TC-GAPDH (indicated by a gain in the FlAsH fluorescence) are illustrated in Figure 3*C*, with the individual fluorescence traces from different trials and their fitted rates shown in Fig. S8, and the mean rates ± SD and calculated extents of heme transfer are reported in Table S1. Figure 3*D* plots the heme transfer rates for each reaction condition as percentages relative to the rate obtained in the simple two-component reaction (set to 100%) run in each independent trial. In general, the rates and estimated extents of heme loss from FlAsH-TC-GAPDH in these reactions matched with what we observed for heme gain into FlAsH-TC-apo-sGCβ in the parallel reactions described in Figure 2. An exception was observed for reactions where Hsp90 or D88N Hsp90 was bound to apo-sGCβ in the absence of ATP, where this caused the rate of heme transfer out from FlAsH-TC-GAPDH to be inhibited by 25% and 35%, respectively (Fig. 3*D* insert). Adding ATP increased the rate of heme transfer from FlAsH-TC-GAPDH by two- to seven-fold when apo-sGCβ was in complex with Hsp90 but not when it was in complex with D88N Hsp90. Because the extents and rates of heme loss from FlAsH-TC-GAPDH correlated with the extent and rates of heme gain into apo-TC-sGCβ under the various reaction conditions, it suggests a highly concerted and efficient heme transfer took place from GAPDH to Hsp90-bound apo-sGCβ in all cases and reveals that bound Hsp90 can mildly decrease or greatly increase the rate of the heme transfer depending on whether ATP is provided and its ATPase activity is intact.

### NO drives heme transfer from GAPDH to apo-sGC**β**

NO stimulates cells to allocate their heme into apo-sGCβ by an unknown mechanism (23). We investigated its mechanism of action in our defined reaction system by using the slow-release chemical NO donor 3-ethyl-3-(ethylaminoethyl)-1-hydroxy-2-oxo-1-triazene (NOC18), whose half-life is reported to be 56 h at pH 7.4 and 22 °C (30). Having various concentrations of NOC18 present in reactions that were initiated by mixing GAPDH–heme complex with FlAsH-TC-apo-sGCβ in the absence or presence of Hsp90/ATP resulted in concentration-dependent and saturable increase in the rate of heme transfer with maximal enhancement observed at 100 μM NOC18 or above in both cases (Fig. S9, *A*–*C*).

**Figure 4:**
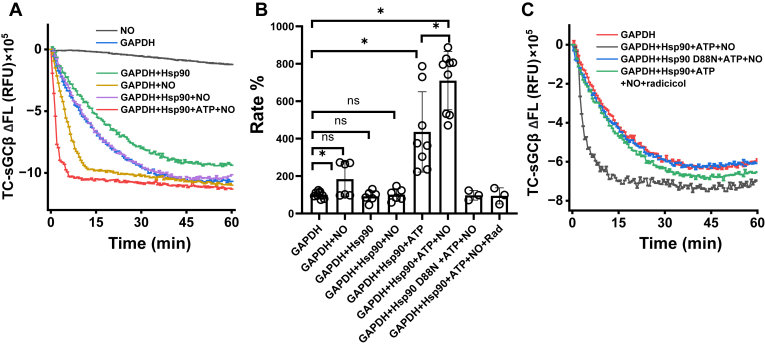
**Effect of NO on GAPDH heme transfer into FlAsH-TC-apo-sGCβ.** Reactions contained 1 μM FlAsH-labeled TC-apo-sGCβ alone or in complex with Hsp90 and were initiated by adding the GAPDH–heme complex to give a final concentration of 1 μM. In some reactions, NOC18, ATP, or and/or radicicol were present or a variant of Hsp90 (D88N) was used as indicated. *A* and *C*, representative fluorescence traces *versus* time (mean ± SD of three replicates) indicating the kinetics and extent of heme gain into FlAsH-TC-apo-sGCβ under the different reaction conditions. *B*, the rates of heme incorporation for each reaction condition as derived by fitting the traces in replica reactions to a single exponential equation. ∗*p* ≤ 0.05, significantly different as indicated.

We went on to test how 100 μM NOC18 may impact heme transfer under our various reaction conditions. Reactions were initiated by mixing GAPDH–heme complex with a FlAsH-apo-TC-sGCβ alone or in complex with Hsp90. Figure 4*A* contains representative fluorescence traces that track the kinetics of heme incorporation under the various indicated reaction conditions, while the individual curves and fitted rates from different trials are shown in Fig. S10 while Table S2 reports the mean rates ± SD. Figure 4*B* plots the heme transfer rates obtained for each reaction condition as percentages relative to the rate obtained in the simple two-component reaction (set to 100%) run in each independent trial. NOC18 added to FlAsH-TC-apo-sGCβ on its own did not change the fluorescence emission *versus* time (Fig. 4*A*). NOC18 added in the two-component reaction caused about a 2-fold increase in the rate of GAPDH heme transfer to FlAsH-TC-apo-sGCβ. In contrast, when NOC18 was added in the three-component reaction (FlAsH-apo-TC-sGCβ in complex with Hsp90), it no longer stimulated the rate of heme transfer unless ATP was also present, in which case the NOC18 addition caused a further doubling of the rate observed for the three-component system plus ATP (Fig. 4, *A* and *B*). The rate enhancement by NOC18 was independent of the amount of GAPDH–heme complex used in the reaction (Fig. S11). Results from experiments that used D88N Hsp90 or included the Hsp90 ATPase inhibitor radicicol (31, 32) (Fig. 4, *B* and *C*) showed that the ability of NOC18 to enhance the rate of heme transfer in the 3-component reaction depended on the ATPase activity of Hsp90.

**Figure 5:**
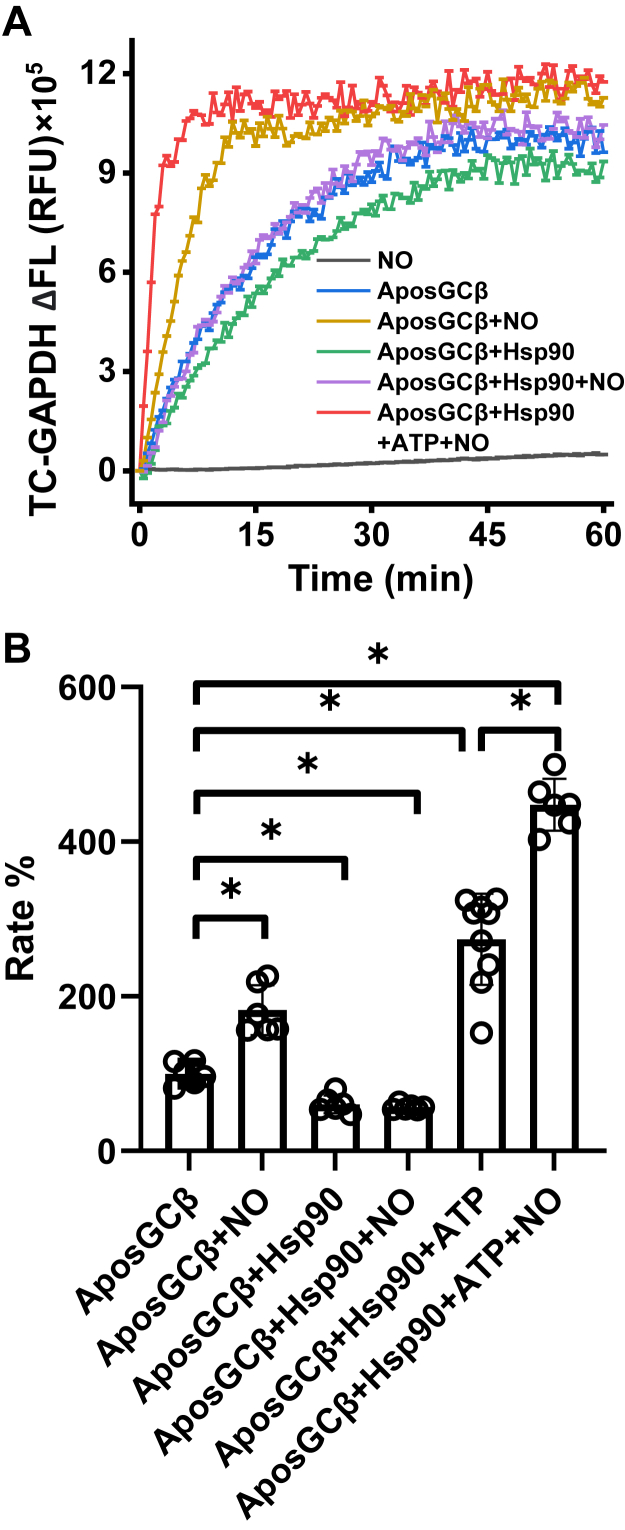
**Effect of NO on heme transfer from FlAsH-TC-GAPDH to apo-sGCβ.** Reactions contained 1 μM of FlAsH-TC-GAPDH-heme complex and were initiated by adding apo-sGCβ either alone or in complex with Hsp90. In some reactions, NOC18 and/or ATP was present as indicated. *A*, representative fluorescence traces (mean ± SD of three replicates) indicating the kinetics and extent of heme loss from FlAsH-TC-GAPDH under the different reaction conditions. *B*, the relative rates of heme incorporation for each reaction condition as derived by fitting the traces in replica reactions to a single exponential equation. ∗*p* ≤ 0.05, significantly different as indicated.

We then studied the effect of NOC18 in parallel reactions where the apo-sGCβ–Hsp90 complex was mixed with a FlAsH-TC-GAPDH-heme complex. Figure 5*A* contains representative fluorescence traces that track heme loss from the FlAsH-TC-GAPDH, with the individual fitted traces being shown in Fig. S12 and the mean rates ± SD being reported in Table S2. Figure 5*B* plots the heme transfer rates for each reaction condition as percentages relative to the rate obtained in the simple two-component reaction (set to 100%) run in each independent trial. In general, the kinetics of FlAsH-TC-GAPDH heme loss mirrored rates we observed when following heme gain into TC-apo-sGCβ. Thus, we conclude that NOC18 could speed a concerted heme transfer from GAPDH to the apo-sGCβ in the two-component reaction but could only speed heme transfer to the apo-sGCβ–Hsp90 complex when ATP was present and the ATPase activity of Hsp90 was intact.

### A ferric to ferrous heme reduction occurs during GAPDH heme transfer into apo-sGC**β**

**Figure 6:**
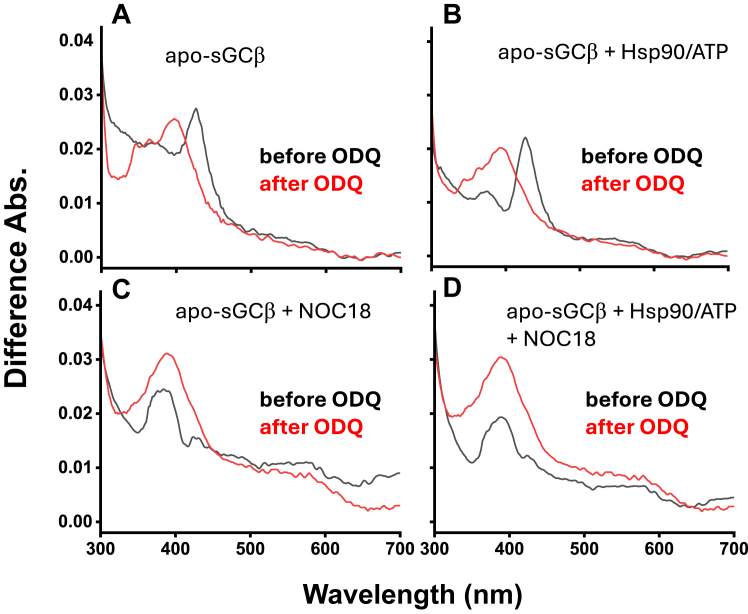
**UV-visible difference spectra indicating ferric heme becomes reduced to ferrous during its transfer from GAPDH into apo-sGCβ.** Reactions contained 2.5 μM apo-sGCβ either alone (*A* and *C*) or with Hsp90 and ATP (*B* and *D*) and were initiated by adding the GAPDH-ferric heme complex (final concentration 2.5 μM GAPDH tetramer containing 1.2 μM heme) and then were run for 30 min in the absence (*A* and *B*) or presence (*C* and *D*) of 100 μM NOC18 at RT. After this 30 min reaction, the samples received ODQ (10 μM), and the reactions were run for a further 30 min. Traces shown are the difference spectra calculated by subtracting the UV-visible spectra recorded at reaction time = 0 from the spectra recorded for the 30 min reaction samples both before (*black traces*) and after (*red traces*) the additional 30 min incubation with ODQ. Traces shown are representative of three independent trials.

To probe mechanism, we first investigated if the heme might undergo a redox change during its transfer from GAPDH into apo-sGCβ, which we have previously observed when free ferric heme is given to purified apo-sGCβ (data not shown). We initiated reactions by mixing a preformed GAPDH–ferric heme complex (final concentration 2.5 μM GAPDH tetramer containing 1.2 μM heme) with 2.5 μM of apo-sGCβ or the apo-sGCβ–Hsp90 complex + ATP, both in the absence or presence of 100 μM NOC18, and recorded UV-visible spectra. Fig. S13, *A*–*D* contain representative spectral traces recorded at 0 and 30 min after initiating the reactions, while Figure 6, *A*–*D* (black traces) contain the resulting difference spectra calculated by subtracting the 0 min spectral trace from the 30 min trace. In reactions without NOC18 (Fig. 6, *A* and *B*), the difference spectra indicate that a sGCβ species with increased Soret absorbance at 427 nm formed after 30 min of reaction in both the absence (Fig. 6*A*) and presence (Fig. 6*B*) of Hsp90/ATP, that indicated ferrous heme sGCβ had formed (33). For the reactions that contained NOC18 (Fig. 6, *C* and *D*), a species with increased absorbance at around 390 nm formed in both the absence (Fig. 6*C*) and presence (Fig. 6*D*) of Hsp90/ATP, consistent with ferric heme transfer from GAPDH resulting in the formation of five-coordinate ferrous heme-NO sGCβ (34). When we then added the sGCβ ferrous heme oxidant 1H-[1,2,4]oxadiazolo[4,3-a]quinoxalin-1-one (ODQ) (35, 36, 37) to the final reaction solutions, it caused the 427 nm heme peak in the difference spectra to shift to 402 nm (Fig. 6, *A* and *B*, red traces) but did not alter the position of the 390 nm Soret peak in the difference spectra for the NOC18-containing samples (Fig. 6, *C* and *D*, red traces). Overall, this behavior is consistent with ODQ oxidizing the sGCβ ferrous heme back to ferric (35) but being unable to oxidize the five-coordinate ferrous heme-NO in sGCβ. Thus, during the 30 min ferric heme transfer from GAPDH into apo-sGCβ, a heme redox transition occurred such that the sGCβ ended up containing either ferrous heme or containing ferrous heme–NO complex if NOC18 was present.

**Figure 7:**
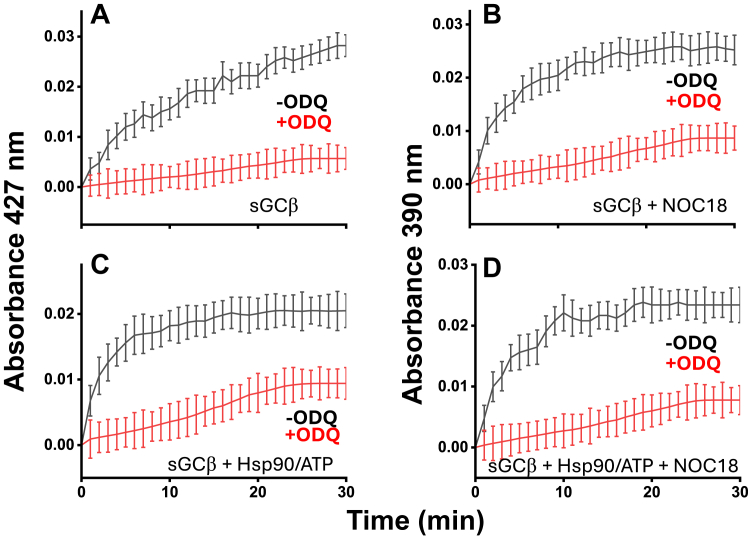
**Having ODQ present during GAPDH heme transfer to apo-sGCβ mutes the spectroscopic changes associated with heme reduction.** Reaction solutions that contained 2.5 μM apo-sGCβ either alone (*A* and *B*) or with Hsp90 and ATP (*C* and *D*), without (*A* and *C*) or with (*B* and *D*) 100 μM NOC18 and with or without 10 μM ODQ as indicated, had GAPDH-ferric heme complex added (final concentration 2.5 μM GAPDH tetramer containing 1.2 μM heme) to initiate the heme transfer reactions which were then run for 30 min while monitoring UV-visible absorbance at 427 nm (*A* and *C*) or 390 nm (*B* and *D*). Absorbance traces shown are the mean ± SD of three replicates and representative of two independent trials.

We next investigated if blocking the ferric to ferrous heme transition would impact heme transfer from GAPDH to apo-sGCβ. To do so, we ran the heme transfer reactions either in the absence or constant presence of ODQ, reasoning that the constant ODQ would prevent ferrous heme from forming and/or building up in the apo-sGCβ. Reactions were initiated by mixing a ferric heme–GAPDH complex with either apo-sGCβ or with apo-sGCβ–Hsp90 complex in the absence or presence of NOC18 and/or ODQ. Figure 7, *A* and *B* contain absorbance traces recorded at 390 or 427 nm *versus* time for two-component reactions that either did or did not contain NOC18, respectively. The continuous presence of ODQ greatly muted the absorbance gains that correspond with either ferrous heme or ferrous heme-NO apo-sGCβ species forming in the reactions. Continuous ODQ had the same impact on the absorbance change *versus* time recorded in the three component reactions (Fig. 7, *C* and *D*). This is consistent with ODQ preventing heme reduction and possibly heme transfer into apo-sGCβ from occurring during the reactions.

**Figure 8:**
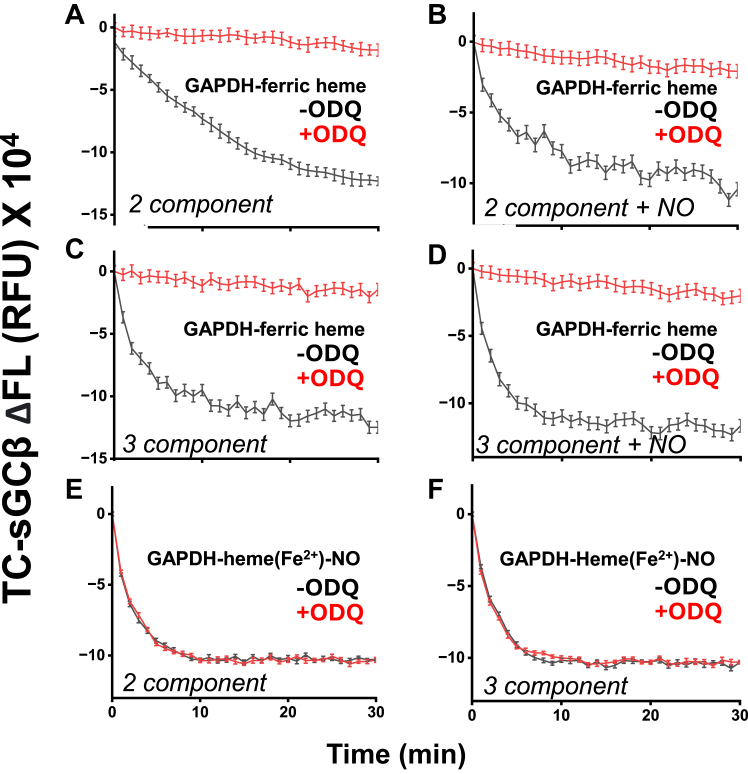
**Having ODQ present diminishes the transfer of ferric heme from GAPDH into FlAsH-TC-apo-sGCβ.** Reaction solutions that contained 1 μM FlAsH-TC-apo-sGCβ alone (*two component*) or with Hsp90 and ATP (*3 component*), with or without 100 μM NOC18 and with or without 10 μM ODQ, had either a GAPDH-ferric heme complex (*A–D*) or a GAPDH ferrous heme-NO complex (*PanelsE* and *F*) added to give 1 μM final concentration to start the transfer reactions. The fluorescence emission was then monitored at RT for 30 min. The traces shown (mean ± SD, three replicates) indicate the kinetics and extent of heme incorporation into FlAsH-TC-apo-sGCβ in the reactions and are representative of two independent trials.

To determine how constant ODQ may impact the extent of heme transfer between GAPDH and apo-sGCβ, we ran reactions as above that utilized FlAsH-TC-apo-sGCβ in order to track its heme incorporation. As shown in Figure 8, *A*–*D*, having ODQ continuously present greatly diminished the fluorescence quenching associated with heme incorporation into the FlAsH-TC-apo-sGCβ in all circumstances (two or three component reactions, ± NOC18), slowing the rates of heme transfer by 94 to 99%. To test if this inhibition by ODQ was due to its ability to oxidize the ferrous heme, we repeated the reactions using a GAPDH containing ferrous heme-NO, which we saw above cannot be oxidized by ODQ. In this case, the continuous presence of ODQ had no effect on the kinetics or extent of ferrous heme-NO transfer from GAPDH into FlAsH-TC-apo-sGCβ (Fig. 8, *E* and *F*). Finally, we checked if ODQ may influence heme release from GAPDH and found that it did not (Fig. S14). Together, the results indicate that a ferric to ferrous transition occurred during heme transfer from GAPDH into apo-sGCβ under all circumstances (*i.e.*, with or without NO or Hsp90/ATP) and this redox transition greatly helped to drive the heme transfer.

### CO antagonizes NO-driven transfer of ferrous heme from GAPDH

**Figure 9:**
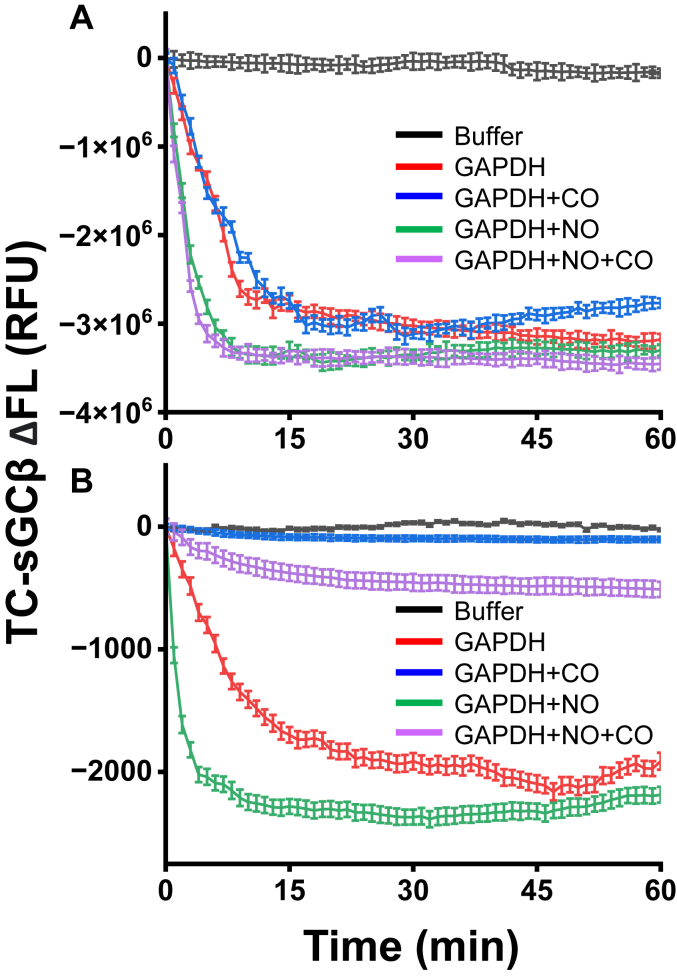
**Effect of CO on the transfer of GAPDH ferrous *versus* ferric heme into FlAsH-TC-apo-sGCβ.** Reactions were run either in air or under an N_2_ atmosphere and were initiated by adding a GAPDH-ferric or GAPDH-ferrous heme complex, respectively, to a FlAsH-labeled TC-apo-sGCβ that was in complex with Hsp90 in the presence of ATP. Some reactions also contained 100 μM NOC18 and/or 500 μM CORM-A (CO donor) as indicated. *A* and *B*, representative fluorescence traces (mean ± SD of three replicates) indicating the kinetics and extent of ferric or ferrous heme transfer into FlAsH-labeled TC-apo-sGCβ under the indicated reaction conditions. Results are representative of two or three independent trials.

Given that NO coordinates to both ferrous or ferric heme iron (38) and that ferric heme in GAPDH exists as a 5-coordinate His-bound or weakly six-coordinate bis-His species (28), it is possible that NO could promote heme transfer by coordinating to the bound heme iron. To test this possibility, we examined if CO, an exclusive ligand for ferrous heme iron (39), would either stimulate the heme transfer on its own or alternatively would antagonize the NO-driven heme transfer from GAPDH to apo-sGCβ. We utilized the water-soluble CO donor CORMA1 to generate CO in our reactions, whose half-life is 2 h at pH 7.4 and 25 °C (40). Reactions were run either in air or under N_2_ atmosphere and were initiated by adding either a preformed ferric or ferrous heme–GAPDH complex, respectively, to a FlAsH-TC-apo-sGCβ–Hsp90 complex + ATP that was either in buffer alone or in buffer containing 100 μM NOC18 alone, 500 μM CORMA1 alone, or containing NOC18 and CORMA1 combined. Figure 9*A* contains fluorescence kinetic traces from representative reactions run in air for the ferric–heme GAPDH complex. In this circumstance, the CORMA1 had no effect on heme transfer into FlAsH-apo-TC-sGCβ in the absence or presence of NOC18. Figure 9*B* shows the fluorescence kinetic traces from representative reactions of the ferrous–heme GAPDH complex run under N_2_ atmosphere. In this circumstance, CORMA1 inhibited ferrous heme transfer from GAPDH into FlAsH-TC-apo-sGCβ on its own and also antagonized the ability of NOC18 to speed the ferrous heme transfer. Thus, CO did not stimulate ferric or ferrous heme transfer and did not inhibit ferric heme transfers from GAPDH in the absence or presence of NO but did inhibit the native and NO-stimulated ferrous heme transfer from GAPDH. This is consistent with NO speeding transfer of ferrous heme from GAPDH by coordinating to the heme iron.

### NO speeds ferric heme dissociation from GAPDH by binding to the heme iron

**Figure 10:**
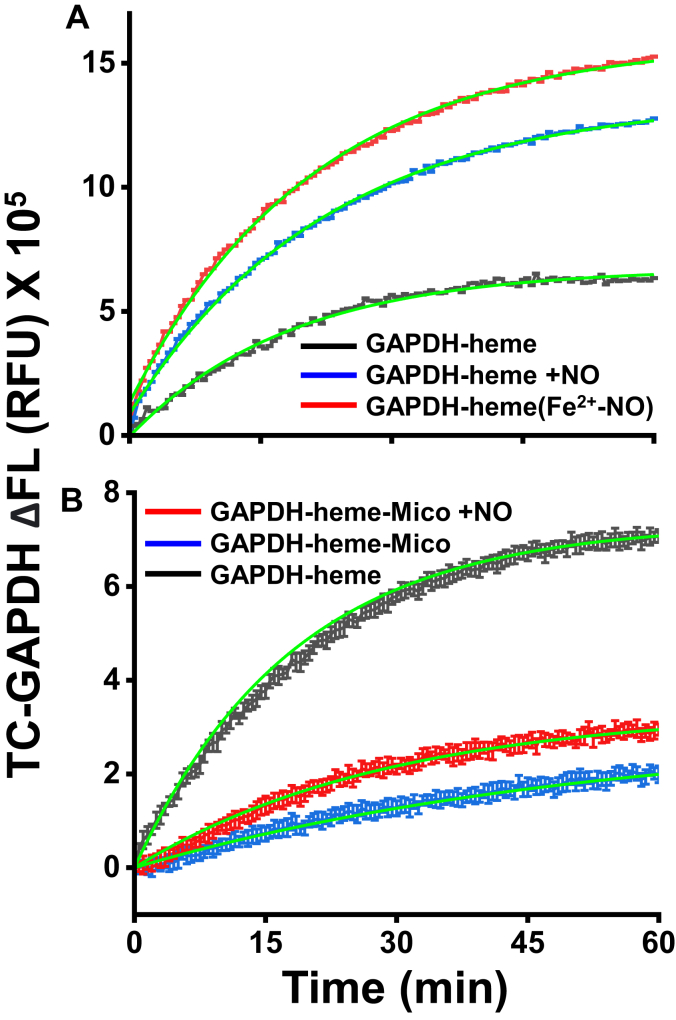
**NO increases the rate of heme dissociation from GAPDH *via* ligation to the heme iron.** Reactions were initiated by mixing either a FlAsH-TC-GAPDH ferric heme complex or a FlAsH-TC-GAPDH ferrous heme-NO complex, with a 30-fold molar excess of GAPDH. In some cases, 100 μM NOC18 or 3 μM miconazole (a ferric iron heme ligand) were also present in the reactions as indicated. *A* and *B*, representative fluorescence traces (mean ± SD, three replicates) indicating the kinetices of heme dissociation from FlAsH-labeled TC-GAPDH under indicated conditions. The fluorescence traces were fit to a single exponential equation (*green line*) to obtain rates and are representative of two or three independent trials.

We next examined how NO may impact the rate of heme dissociation from GAPDH. We followed dissociation of the FlAsH-TC-GAPDH-ferric heme complex after mixing it with a 30-fold excess of GAPDH in the absence or presence of 100 μM NOC18 and also examined if NOC18 would impact the dissociation of a FlAsH-TC-GAPDH heme complex when miconazole had been bound to its ferric heme to form a stable six-coordinate complex (Fig. S15), reasoning that this should antagonize NO binding with the ferric heme iron. Heme dissociation rates in all cases were followed by the gain in FlAsH-TC-GAPDH fluorescence. The kinetic traces in Figure 10*A* show that NOC18 increased the rate of ferric heme dissociation from GAPDH by 2-fold (0.04 ± 0.01 min^−1^ *versus* 0.08 ± 0.01 min^−1^), such that it nearly matched the faster dissociation rate of a preformed GAPDH ferrous heme–NO complex. When miconazole was bound to the GAPDH ferric heme, it decreased the rate of heme release by 3-fold (0.054 ± 0.009 min^−1^ *versus* 0.019 ± 0.008 min^−1^) and in this circumstance, the NOC18 was almost completely unable to increase the rate of heme dissociation (Fig. 10*B*). Together, this implies that NO increases the rate of ferric heme release from GAPDH by it coordinating to the heme iron.

### Nitrosation of protein Cys residues is not involved in the NO-driven heme transfer

Because NO exposure can lead to S-nitrosation of protein Cys residues (SNO modifications) (41) and SNO buildup in GAPDH is known to inhibit heme allocation in cells (42), we investigated if SNO modifications occurred in GAPDH or apo-sGCβ during a heme transfer reaction run in the presence of 100 μM NOC18. As shown in Fig. S16, SNO build up did not occur in GAPDH but did occur in sGCβ after 30 min or longer of NOC18 exposure. This delay period is at least three times longer than the time it takes to complete the heme transfer in this reaction condition (see Fig. 4). As a second approach, we investigated if NOC18 would still speed the rate of heme transfer from GAPDH to FlAsH-TC-apo-sGCβ when we utilized a GAPDH variant whose three Cys residues have all been substituted by Ser, thus preventing any SNO modifications. NOC18 sped heme transfer from this GAPDH variant to a normal extent (Fig. S17). Together, these data rule out a role for protein SNO modifications in how NOC18 speeds heme transfer in our reactions.

## Discussion

GAPDH supplies heme to the apo-sGCβ subunit during its functional maturation in cells. Inserting a TC sequence allowed us to follow heme transfer out of FlAsH-TC-GAPDH or into FlAsH-TC-apo-sGCβ to investigate the mechanism and regulation of their heme transfer. Overall, we found that most of the heme bound in GAPDH (73–100%) could transfer into apo-TC-sGCβ and that the rate of heme transfer out from TC-GAPDH was coupled to the rate of heme transfer into TC-apo-sGCβ under all reaction conditions. The heme transfer relied on direct contact between the GAPDH–heme complex and apo-sGCβ and did not involve simple heme dissociation into solution. Indeed, we saw that GAPDH held fast to its heme when its recipient partner was an apo-sGCβ variant that could not accept the heme, despite this variant being able to bind normally with GAPDH. Overall, this suggests a mechanism whereby the GAPDH heme complex interacts with apo-sGCβ to engage in a direct protein-to-protein heme transfer that is tightly coupled regarding its rate and extent.

### Heme undergoes a redox transition that helps to drive its transfer into apo-sGC**β**

Our results utilizing spectroscopy and ODQ revealed that ferric heme transfer from GAPDH into apo-sGCβ was accompanied by heme reduction to the ferrous state under all our reaction conditions (*i.e.*, two- or three-component system, ± NO). The fact that the transfer of ferrous or ferric heme from GAPDH to apo-sGCβ occurred at similar rates (see Fig. 2) indicates that the heme reduction step was not rate limiting under our reaction conditions. Surprisingly, the heme reduction occurred despite our buffers and protein solutions not containing low molecular weight reductants like DTT or β-mercaptoethanol. This implies that the heme-reducing equivalent comes from a Cys thiol in either the GAPDH or apo-sGCβ proteins. Such thiol-mediated heme reduction is not unprecedented (43, 44, 45). Because we found that substituting all three Cys residues to Ser in GAPDH had no effect on the heme transfer, the reducing equivalent is likely supplied by one or more of the nine Cys residues present in apo-sGCβ. Having the apo-sGCβ protein provide an electron to the heme would be consistent with the following: (i) the rates of the heme redox transition during heme transfer (as followed by UV-visible spectral changes, Fig. 7) not exceeding the rates of heme incorporation into FlAsH-TC-apo-sGCβ (as followed by fluorescence, Fig. 8); (ii) CO being unable to block ferric heme transfer from GAPDH despite it being able to block the ferrous heme transfer; (iii) the established need for sGCβ Cys residues in generating a heme-containing functional sGC (46, 47). Importantly, when the heme redox transition was antagonized by ODQ, it severely compromised the heme transfer under all reaction conditions (2 and 3-component, ± NO). Thus, a heme reduction step appeared to be universally involved and important to enable the ferric heme transfer from GAPDH into apo-sGCβ. This is consistent with the thermodynamics of sGCβ heme binding favoring the ferrous heme over the ferric form (48). The mechanism and biological relevance of the heme reduction step uncovered here can now be investigated.

### Heme transfer from GAPDH to apo-sGC**β** displays Hsp90/ATP dependence

Our three-component reaction system provided a way to explore Hsp90 function in GAPDH heme transfer to apo-sGCβ, which had previously been studied only in cell culture settings (10, 11, 14). In cells, Hsp90 binding to apo-sGCβ is strictly required for the heme allocation, and even when the Hsp90 is bound, no heme allocation will occur if the ATPase activity of the Hsp90 is blocked by pharmacologic inhibitors like radicicol or by point mutagenesis (D88N) (11, 14). An exact recapitulation of this cellular behavior in our *in vitro* reaction system would have had no heme transfer occurring from GAPDH to apo-sGCβ in the absence of Hsp90 and ATP or perhaps would have had the bound Hsp90 blocking the heme transfer from GAPDH into apo-sGCβ unless ATP was present. In our reactions, we found that GAPDH could transfer heme to apo-sGCβ in the absence of Hsp90 and that having Hsp90 bound to apo-sGCβ in the absence of ATP inhibited the rate of heme transfer by only 10 to 15% and by 25% when the D88N Hsp90 was bound. It is unclear what enables the “Hsp90-independent” heme transfer from GAPDH to apo-sGCβ to occur in the *in vitro* reaction system, and perhaps more importantly, what prevents such “Hsp90-independent” GAPDH heme transfer to apo-sGCβ from happening inside living cells. Regarding the latter question, possibilities include the relative scarcity of heme-bound GAPDH inside mammalian cells (estimated to be one heme bound per five GAPDH tetramers) (19) compared to the one to one or greater GAPDH-heme to apo-sGCβ stoichiometry used in our reactions. Also, Hsp90 co-chaperones (49) or posttranslational modifications (50) in cells might enable the bound Hsp90 to better block heme transfer into apo-sGCβ in the absence of ATP. In any case, our findings imply that in intact cells, an additional kinetic barrier exists to block heme transfer from GAPDH to apo-sGCβ. The three-component reaction system can now be deployed to identify its nature.

We found that Hsp90 increased the rate of heme transfer from GAPDH to apo-sGCβ only in the presence of ATP. Experiments with the ATPase inhibitor radicicol or the ATPase-defective Hsp90 D88N variant confirmed that the ATP-based rate enhancement was tied to ATP being hydrolyzed by Hsp90. This implies that ATP activation of Hsp90 conformational cycling (51, 52), which enables the end-stage folding or small molecule incorporation of its client proteins (51), also enables Hsp90 to speed heme transfer into apo-sGCβ in our reactions. We previously established that Hsp90 binding to apo-sGCβ, on its own, stabilizes apo-sGCβ in alternative conformations that may be more amenable for heme delivery (10, 27). Our current results imply that the change due to Hsp90 binding alone is insufficient and additionally requires ATP-driven conformational cycling to speed heme transfer into apo-sGCβ.

### NO acts by speeding heme release from GAPDH

NO caused a two-fold increase in the rate of ferric heme transfer from GAPDH into apo-sGCβ in the two-component reaction. This correlated with it causing a two-fold increase in the rate of ferric heme dissociation from GAPDH, which in turn relied on NO being able to coordinate to the heme iron. This implies that NO coordination to the bound ferric heme reduces its affinity toward GAPDH. NO appears to have the same kinetic effect on bound ferrous heme, based on our finding that the ferrous heme–NO complex dissociates faster from GAPDH. Given that we could discount NO-derived SNO modifications in GAPDH or apo-sGCβ being involved, we conclude that NO speeds heme transfer from GAPDH into apo-sGCβ by binding to the heme iron and speeding the rate of heme dissociation from GAPDH.

### The kinetic effect of NO is beholden to ATP hydrolysis by Hsp90

In our 3-component reactions that used Hsp90-bound apo-sGCβ, we found that NO could no longer speed the heme transfer from GAPDH unless ATP was present and could be hydrolyzed by the bound Hsp90. This uncovered a clear inhibitory effect of the bound Hsp90. If we assume that NO speeds the heme transfer by causing faster GAPDH heme release (as noted above), then having the bound Hsp90 prevent NO from speeding the heme transfer into apo-sGCβ implies that when ATP-driven Hsp90 conformational cycling is not possible, the Hsp90 holds apo-sGCβ in conformations that limit its rate of heme intake from GAPDH such that it cannot be increased by NO. Presumably, ATP hydrolysis by the bound Hsp90 removes this kinetic barrier by enabling apo-sGCβ to adopt alternative conformations that allow it a faster heme uptake and this in turn allows the positive kinetic effect of NO on GAPDH heme release to manifest as a faster heme incorporation into the apo-sGCβ. These concepts reveal how NO and Hsp90 work by different mechanisms that combine to regulate heme transfer from GAPDH into apo-sGCβ.

### New insights on the mechanism and regulation of GAPDH heme transfer to apo-sGC**β**

**Figure 11:**
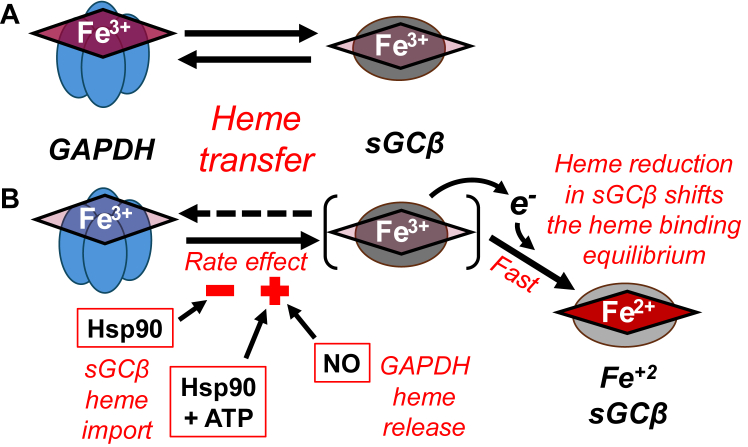
**Ferric heme transfer from GAPDH into apo-sGCβ is regulated by a heme redox transition, NO, and Hsp90/ATP *via* distinct mechanisms.** The transfer of ferric heme from GAPDH into apo-sGCβ is tightly coupled regarding its rate and extent, relies on protein–protein contact, and results in reduction of the ferric heme to ferrous inside apo-sGCβ. *A*, heme transfer occurs to a minor extent in the absence of heme reduction. *B*, the heme redox transition greatly drives the equilibrium toward the right and increases the observed extent and rate of the heme transfer. In this circumstance, NO and Hsp90 can speed heme transfer by two different mechanisms: NO acts by binding to the ferric heme iron in GAPDH to increase its rate of release, while Hsp90 speeds heme intake into the apo-sGCβ by causing protein conformational changes that are driven by its ATP hydrolysis. In all cases, the extent and rate of heme transfer from GAPDH is determined by the ability of the apo-sGCβ to accept the heme.

Our findings are summarized in Figure 11. Ferric heme transfer out of GAPDH is tightly coupled to and dependent on heme intake into apo-sGCβ regarding its rate and extent, and the heme transfer is poor if the ferric heme cannot undergo reduction to its ferrous state inside the apo-sGCβ recipient. This reveals a poor driving force exists for ferric heme transfer between the two proteins, and in a circumstance where the heme reduction can be quickly reversed (*i.e.*, in the presence of ODQ), neither Hsp90/ATP nor NO can impact the rate of ferric heme transfer. However, when the ferric to ferrous heme transition can occur, it drives the heme-binding equilibrium toward the right and this increases the extent and observed rate of the heme transfer. Under this circumstance, the kinetic effects of NO and Hsp90 can manifest by two different mechanisms: NO acts by binding to the GAPDH ferric heme iron to increase its rate of release from GAPDH, while Hsp90 governs the rate of heme intake into apo-sGCβ by supporting protein conformational changes that can have either negative or positive kinetic impacts depending on ATP hydrolysis by the Hsp90. In all cases, the rate and extent of the heme transfer is controlled by the ability of the apo-sGCβ to accept the heme, which depends on it having a functional heme-binding site, being able to support a ferric to ferrous heme transition, and it being in an amenable conformation.

### Biological importance

Heme incorporation into the apo-sGCβ subunit enables a functional sGCα/β heterodimer to form that can catalyze production of cGMP, a second messenger molecule that is involved in a myriad of biological functions (53). Our finding that a ferric to ferrous heme transition in apo-sGCβ helps to drive its heme acquisition from GAPDH and that the rate of heme insertion into apo-sGCβ is governed by Hsp90 through its ATP-dependent conformational cycling, provide new insight on how the supply of heme to apo-sGCβ in cells may occur and be regulated. Our finding that NO speeds heme dissociation from GAPDH likely helps explain how NO exposure causes cells to begin redistributing their GAPDH-bound heme into their apo-sGCβ subpopulation (23). Because the population of apo-sGCβ is estimated to be 30 to 80% of the total sGCβ due to a natural heme deficit that exists in cells and tissues (21), the mechanisms we uncovered here are likely to help explain how the functional sGCα/β heterodimer forms in cells and how its level and consequent cGMP production can be regulated in biological settings.

## Experimental procedures

### General methods and materials

The TC-FlAsH Tetra cysteine Tag Detection Kit was obtained from Invitrogen. All other reagents and materials were obtained from source reported elsewhere (14, 17). Generation and characterization of the ferrous heme-NO reagent was reported previously (18).

### Molecular biology

pET20b expression plasmid containing rat sGCβ1(1–385) with a C-terminal His_6_ tag (sGCβ) was a gift from Dr Michael Marletta (University of California). Expression plasmids containing rat sGCβ(1–385) with a TC motif (CCPGCC) inserted at residue 239 to 244 (TC-sGCβ(1–385)) (17), TC-sGCβ(1–385) Y135A R139A (17), human GAPDH containing a TC motif to bind FlAsH (TC-GAPDH) (19), and human heat shock protein 90β (Hsp90) (14) were reported previously.

### Protein purification

His_6_-tagged apo-sGCβ and mutants, full length human Hsp90, and C-terminal GST-tagged versions of human GAPDH or TC-GAPDH were expressed in *Escherichia coli* BL21(DE3) and purified using previously reported methods (17, 27, 42). The GST tag was removed enzymatically from the GAPDH proteins during the purification process. All the purified proteins were concentrated and stored at −80 °C in PBS buffer (137 mM NaCl, 2.7 mM KCl, 10 mM Na_2_HPO_4_, and 1.8 mM KH_2_PO_4_.), pH 7.4.

### FlAsH labeling of TC-sGC**β** and TC-GAPDH proteins

TC-sGCβ and TC-GAPDH proteins were labeled with FlAsH using a method reported previously (17) and were run through a PD-10 column prior to use.

### GAPDH ferric or ferrous heme complex generation

GAPDH–heme complexes were generated using methods reported previously (28). Briefly, GAPDH was incubated with ferric heme for 15 min at room temperature at a 1 to 1.1 heme to GAPDH tetramer ratio and then passed through a PD-10 column (GE). The GAPDH ferrous heme–NO complex was made by the same procedure using reagent ferrous heme-NO. The GAPDH–ferric heme complex was reduced to ferrous by adding dithionite under an N_2_ atmosphere as reported previously (54).

### Monitoring heme transfer into TC-apo-sGC**β** and out of TC-GAPDH

Heme transfer from GAPDH heme complex into FlAsH-TC-apo-sGCβ proteins was monitored in reactions as described previously with modification (41). Briefly, reactions were run at room temperature in black masked 96-well fluorescence plates. Wells contained PBS, pH7.4 and 1 μM of FlAsH-TC-apo-sGCβ. GAPDH–heme complex was added to start the reaction, and the fluorescence emission was monitored over time in an iD5 max fluorescence microplate reader (Molecular Devices) with excitation/emission set at 490 nm and 530 nm. In the same way, the rates of ferric or ferrous heme-NO loss from pre-made FlAsH-TC-GAPDH complexes was determined in parallel by mixing apo-sGCβ into reaction solutions that contained 1 μM of FlAsH-TC-GAPDH-heme tetramer to start the reactions.

To monitor the kinetics of ferrous heme transfer from GAPDH into FlAsH-TC-apo-sGCβ, reactions were run in the same solutions described above at room temperature in anaerobic fluorescence cuvettes. Solutions of FlAsH-TC-apo-sGCβ or GAPDH ferric heme complex separately underwent repeated vacuum and N_2_ cycling using a gas train and put under N_2_ atmosphere (54). The GAPDH ferric heme complex was reduced to ferrous by dithionite addition and was transferred anaerobically into a fluorescence cuvette containing 1 μM FlAsH-TC-apo-sGCβ. The fluorescence emission was monitored *versus* time using a SF-2500 Fluorescence Spectrophotometer (Hitachi) with excitation/emission set at 490 nm and 530 nm. In the same way, the rates of ferrous heme loss from FlAsH-TC-GAPDH were determined in parallel reactions by transferring apo-sGCβ into an anerobic cuvette containing 1 μM of FlAsH-TC-GAPDH-ferrous heme tetramer.

### Difference spectra to assess heme transfer and heme redox state

UV-visible spectra were obtained using a Shimadzu UV-2401 PC spectrophotometer. Spectra were recorded at room temperature for solutions of 2.5 μM GADPH tetramer that contained ferric heme or ferrous heme-NO, with spectra being recorded before and 30 min after adding 2.5 μM apo-sGCβ. Difference spectra were obtained by subtracting the original spectra from the second one. In some cases, ODQ (10 μM) was added to the GADPH ferric heme or ferrous heme-NO solution before adding in the apo-sGCβ or was added to the solution after the second spectrum had been obtained at 30 min, and in this case, another spectrum was recorded after an additional 30 min.

### Determining the rate of GAPDH-heme dissociation

Solutions containing 1 μM of FlAsH-TC-GAPDH tetramer containing either ferric heme or ferrous heme-NO were distributed into a 96-well plate and then had GAPDH tetramer added to give 30 μM. The fluorescence intensity was then monitored immediately after mixing over the next 60 min in an iD5 max fluorescence microplate reader (Molecular Devices) with excitation/emission set at 490 nm and 530 nm. In some cases, the initial solution also contained 3 μM miconazole and/or 10 μM ODQ and/or 100 μM NOC18.

### Fluorescence polarization measurements

Residual fluorescence polarization measurements on FlAsH-labeled TC-apo-sGCβ or TC-GAPDH were performed at 25 °C using methods described previously (27).

### Detection of Cys S-nitrosation in sGC and GAPDH using the biotin switch assay

The biotin switch assay was performed as described previously (55) with modifications. Briefly, solutions contained TC-sGCβ and GAPDH (10 μM each) with or without 50 μM Hsp90 in 250 mM Hepes buffer, pH 7.7, and were incubated with 100 μM NOC18. Sample aliquots were taken from the reaction mixtures at different times and put on ice. Sample aliquots were then incubated with 10 mM methylmethane thiosulfonate, 2% SDS, 1 mM EDTA, and 0.1 mM neocuproine at 50 °C for 20 min and then passed through a PD10 gel filtration column to remove NOC18 and methylmethane thiosulfonate. The eluted samples were then incubated with 10 mM EZ-Link HPDP-Biotin (Thermo Fisher Scientific) and 1 mM ascorbate for 1 h at room temperature to label protein Cys-NO groups with biotin. Samples were subjected to SDS-PAGE Western blot analysis as reported previously (55).

### Statistics and data analysis

Data analysis was performed using Origin Lab 2022 or GraphPad Prism 10 (Dotmatics). Sample replicates were either in triplicate or duplicate and were fixed before data were observed. Experiments were done in two or three independent trials as indicated in the figure legend. Apparent heme transfer rates were calculated using a nonlinear regression analysis with exponential model in OriginLab 2022. The fluorescence kinetic traces for FlAsH-TC-apo-sGCβ were fit as single exponential and for FlAsH-TC-GAPDH were fit as single or double exponential. The UV-vis absorbance kinetic traces were fit as a single exponential. In some cases, to minimize the variability in the absolute rates obtained between the individual trials for each individual reaction condition, we set as 100% the mean rate observed for each trial's reaction that contained GAPDH-heme and apo-sGCβ and then used this to convert the absolute rates obtained for all the other reaction conditions in the same trial to percentages. This allowed us to pool and graph together results from the individual trials. GraphPad Prism 10 was used to evaluate the differences between groups by a one-way ANOVA, and *p* < 0.05 was considered statistically significant in all analyses.

## Data availability

All data are contained within the article or available upon request.

## Supporting information

This article contains supporting information.

## Conflict of interest

The authors declare that they have no conflicts of interest with the contents of this article. The content is solely the responsibility of the authors and does not necessarily represent the official views of the National Institutes of Health.
