# Supplementary material for: Heme delivery into soluble guanylyl cyclase requires a heme redox change and is regulated by NO and Hsp90 by distinct mechanisms
Source: J Biol Chem. 2025 Feb 13;301(3):108315. doi: 10.1016/j.jbc.2025.108315 (PMC11938259; doi:10.1016/j.jbc.2025.108315)
Supplement: Supporting material [file mmc1.pdf]

Heme Delivery into Soluble Guanylyl Cyclase Requires a Heme Redox Change and is Regulated by NO and Hsp90 by Distinct Mechanisms

Yue Dai and Dennis J. Stuehr*

**Number**	**Page number**
Table S1	S-2
Table S2	S-3
Fig. S1	S-4
Fig. S2	S-5
Fig. S3	S-6
Fig. S4	S-7
Fig. S5	S-8
Fig. S6	S-9
Fig. S7	S-10
Fig. S8	S-11
Fig. S9	S-12
Fig. S10	S-13
Fig. S11	S-14
Fig. S12	S-15
Fig. S13	S-16
Fig. S14	S-17
Fig. S15	S-18
Fig. S16	S-19
Fig. S17	S-20

Table S1. Fitted rates of heme insertion from GAPDH ferric heme complex into FlAsH-TC-apo-sGCβ. Fluorescence traces used in Fig. 3A, 3C, 4A, 4B were fitted with single exponential model and the mean rates +/- S.D. are listed. The heme transfer percentages were calculated using 64% of quenching equaling 100% heme insertion into FlAsH-TC-apo-sGCβ.

TC-sGCβ sample Group	*k*(min^{-1})	*Transferred heme %*	*n*
GAPDH	0.09±0.03	96±10	18
GAPDH + Hsp90	0.10±0.04	88±8	15
GAPDH + Hsp90 D88N	0.06±0.02	92±3	9
GAPDH + Hsp90 + ATP	0.44±0.07	94±8	9
GAPDH + Hsp90 D88N + ATP	0.05±0.01	87±3	9
GAPDH + ATP	0.05±0.01	90±7	3
GAPDH + NO	0.20±0.09	99±11	6
GAPDH + Hsp90 + NO	0.11±0.02	93±8	6
GAPDH + Hsp90 + ATP + NO	0.62±0.09	105±12	9
GAPDH + Hsp90 D88N + ATP + NO	0.07±0.02	98±8	3
GAPDH + Hsp90 + ATP + NO + Radicicol	0.07±0.03	90±5	3

Table S2. Fitted rates of heme loss from FlAsH-TC-GAPDH during heme transfer to apo-sGCβ. Fluorescence traces used in Fig. 3B, 3D, 5A were fitted with double exponential model and the mean rates +/- S.D. are listed. The heme transfer percentage were calculated by setting the initial fluorescence of the FlAsH-TC-GAPDH-heme complex as 78% of the fluorescence for heme-free FlAsH-TC-GAPDH.

TC-GAPDH sample group	k_1 *(min^{-1})*	k_2 *(min^{-1})*	*A1:A2*	*Transferred heme%*	*n*
Apo-sGCβ	0.12±0.03	0.018±0.01	60:40	82±3	18
Apo-sGCβ + Hsp90	0.08±0.03	0.0035±0.002	60:40	79±12	15
Apo-sGCβ + Hsp90 D88N	0.07±0.03	0.002±0.0001	50:50	75±8	9
Apo-sGCβ + Hsp90 + ATP	0.52±0.05	0.010±0.002	80:20	94±9	9
Apo-sGCβ + Hsp90 D88N + ATP	0.06±0.04	0.0001±0.0001	50:50	80±10	9
Apo-sGCβ + ATP	0.09±0.01	0.0001±0.0001	60:40	91±6	3
Apo-sGCβ + NO	0.24±0.05	0.010±0.005	80:20	91±7	6
Apo-sGCβ + Hsp90 + NO	0.07±0.01	0.0001±0.0001	40:60	73±20	6
Apo-sGCβ + Hsp90 + ATP + NO	0.85±0.07	0.0001±0.0001	95:5	94±11	6

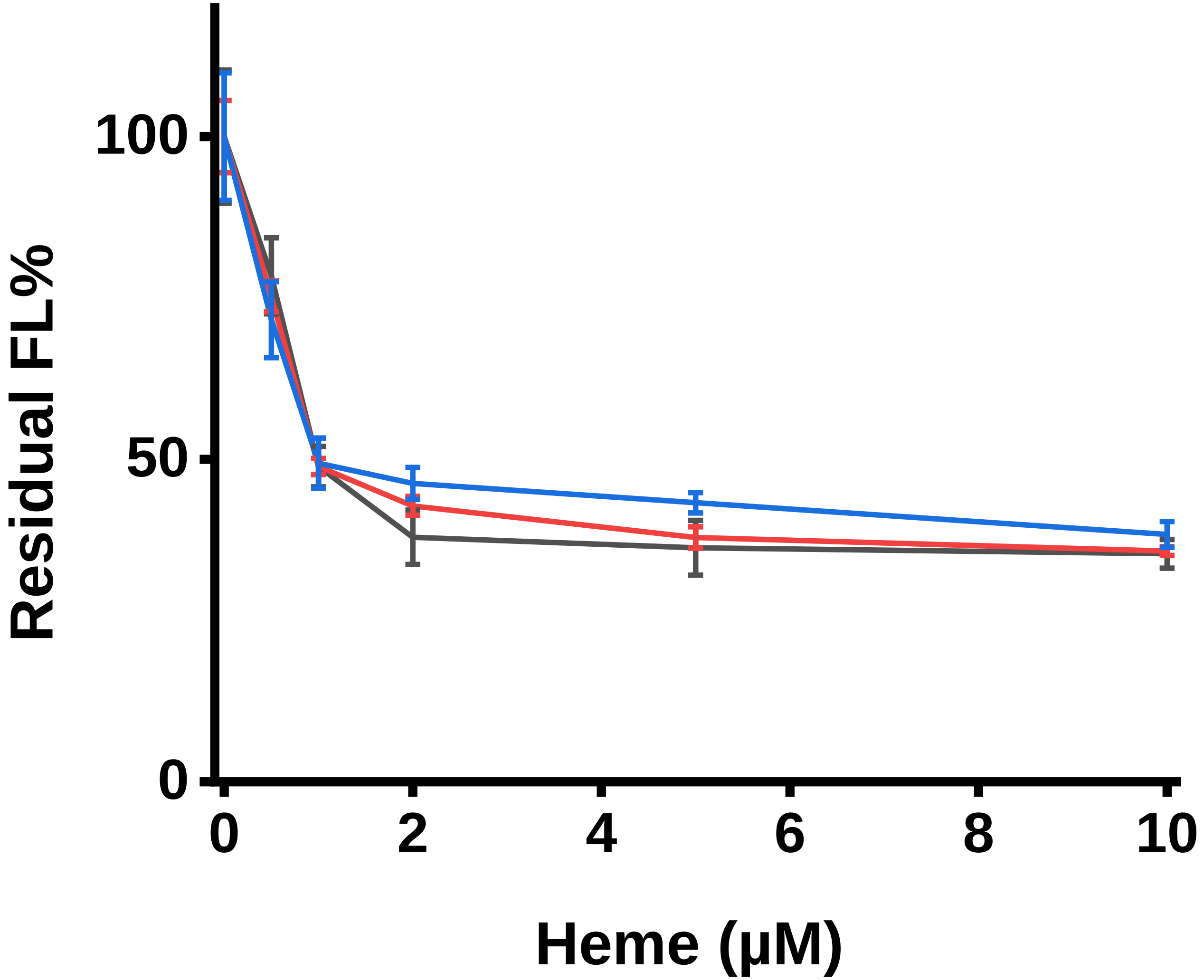

Fig. S1. Heme titration of FlAsH-TC-apo-sGCβ under various conditions. FlAsH-TC-apo-sGCβ (1 μM) either alone (black) or bound with Hsp90 in the absence (red) or presence (blue) of ATP was titrated with increasing amounts of ferric heme and the FlAsH fluorescence emission monitored. Data are the mean +/- SD for three replicates.

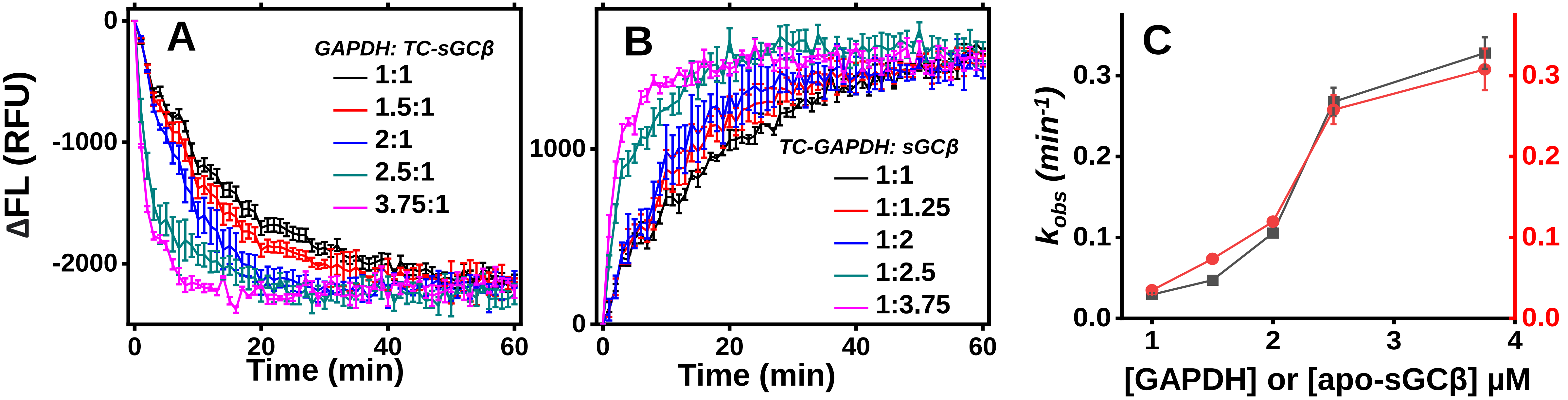

Fig. S2. The kinetics of heme incorporation into apo-sGCβ and heme loss from GAPDH are tightly coupled. Panels *A and B*- Heme transfer reactions between GAPDH-heme tetramer (approx. 1 heme per tetramer) and apo-sGCβ were run at different concentration ratios (in μM) as indicated in the panels, and the fluorescence emission from either FlAsH-TC-apo-sGCβ (*A*) or FlAsH-TC-GAPDH (*B*) was recorded versus time. Panel *C*- The rates obtained by fitting the fluorescence traces in Panel *A* (red) and *B* (black) to a single exponential equation were plotted versus the molar ratio of the two reacting proteins.

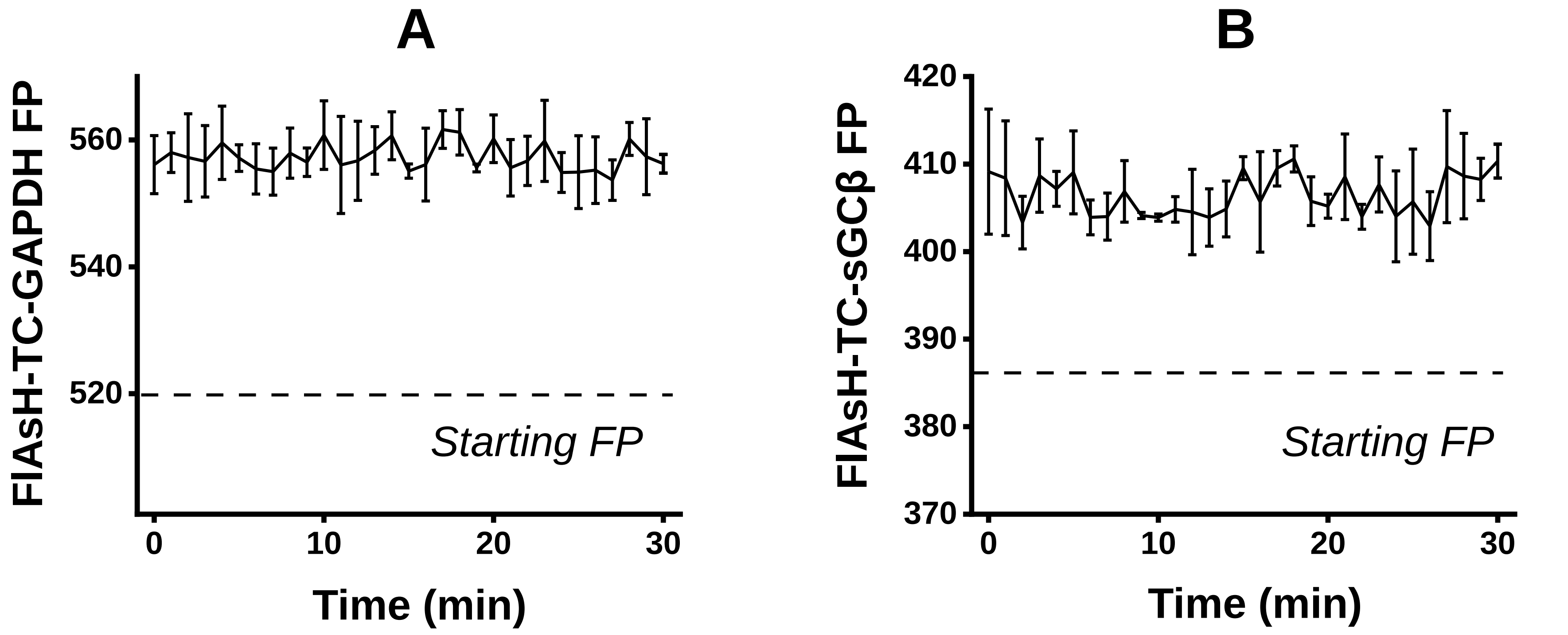

Fig. S3. GAPDH-heme and apo-sGCβ rapidly form a complex upon mixing. Wells contained 1 μM each of (A) FlAsH-TC-GAPDH or (B) FlAsH-TC-apo-sGCβ. The residual fluorescence polarization was recorded before (starting FP) and then every min after the respective unlabeled partner protein was added. The solid lines plot the mean +/- SD values for three replicates and are representative of two independent trials.

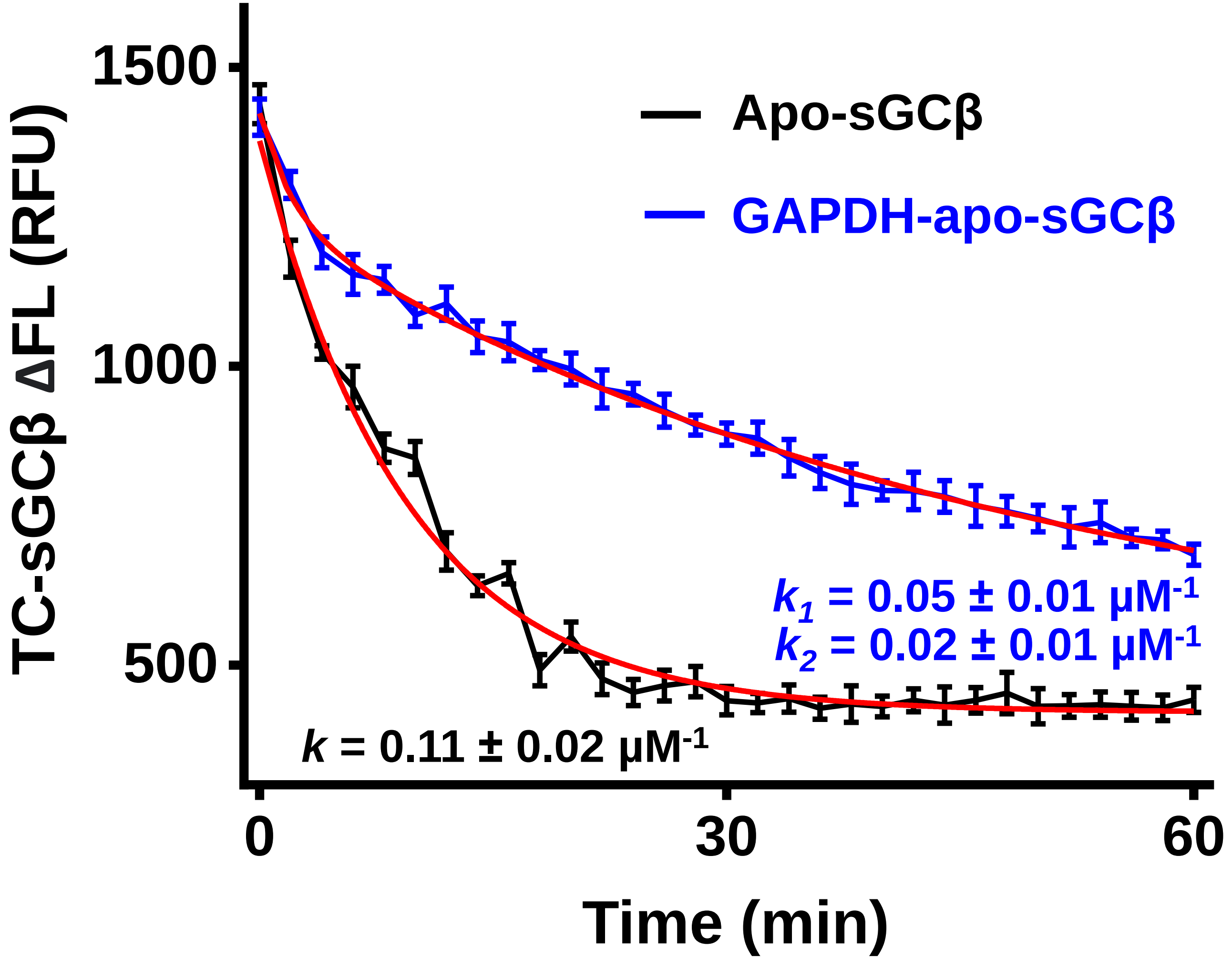

Fig. S4. Pre-binding GAPDH to FlAsH-TC-apo-sGCβ slows its import of heme from a GAPDH-heme complex. A GAPDH-heme complex was mixed with FlAsH-TC-apo-sGCβ either alone (apo-sGCβ) or after it had been incubated with GAPDH to form a complex (Apo-sGCβ + GAPDH) and the FlAsH fluorescence emission was recorded every 2 min for 60 min. Data are the mean +/- SD for three replicates and are representative of two trials. Red traces indicate the lines of best fit according to a one or two-exponential equation to yield the rates indicated in the panel.

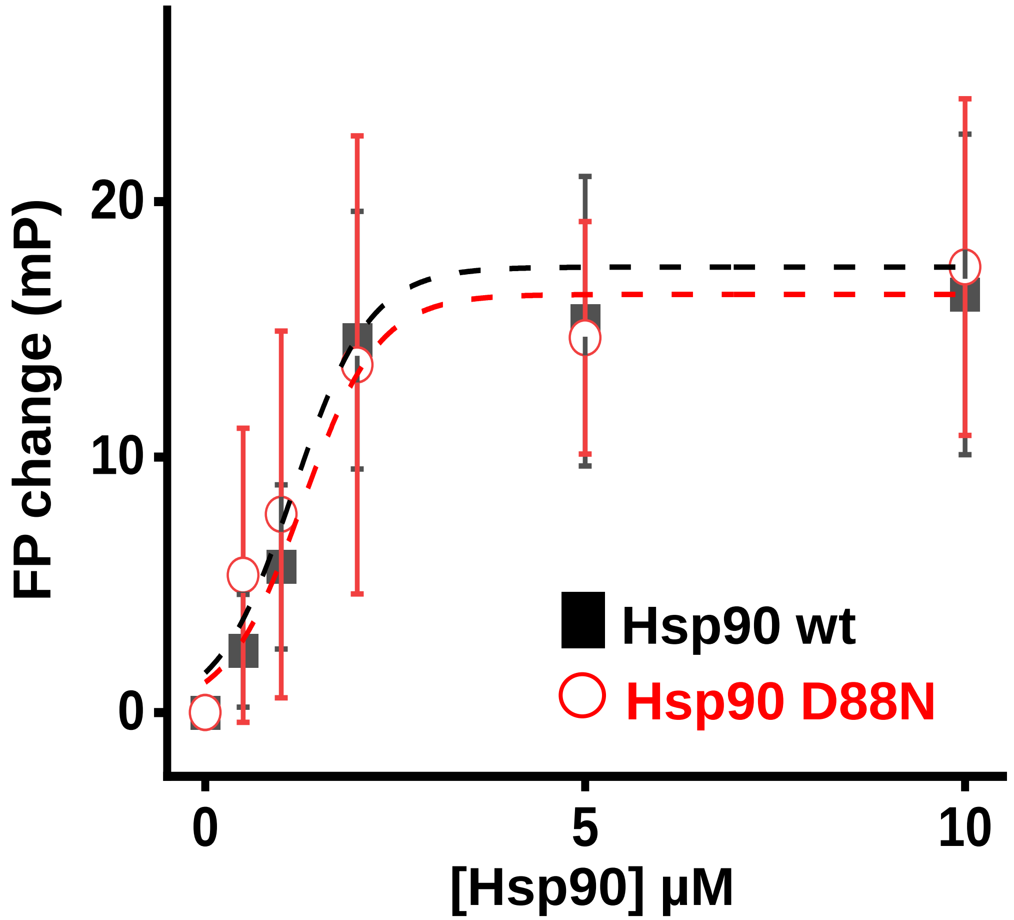

Fig. S5. Complex formation between FlAsH-TC-apo-sGCβ and wild type Hsp90 or D88N Hsp90 as a function of their concentration. Wells containing 0.5 μM FlAsH-TC-apo-sGCβ had wild-type or D88N Hsp90 added at the indicated concentrations and the FlAsH residual fluorescence polarization in the samples was recorded after incubating the mixtures for 30 min. The dashed lines plot the mean +/- SD values for three replicates and are representative of two independent trials.

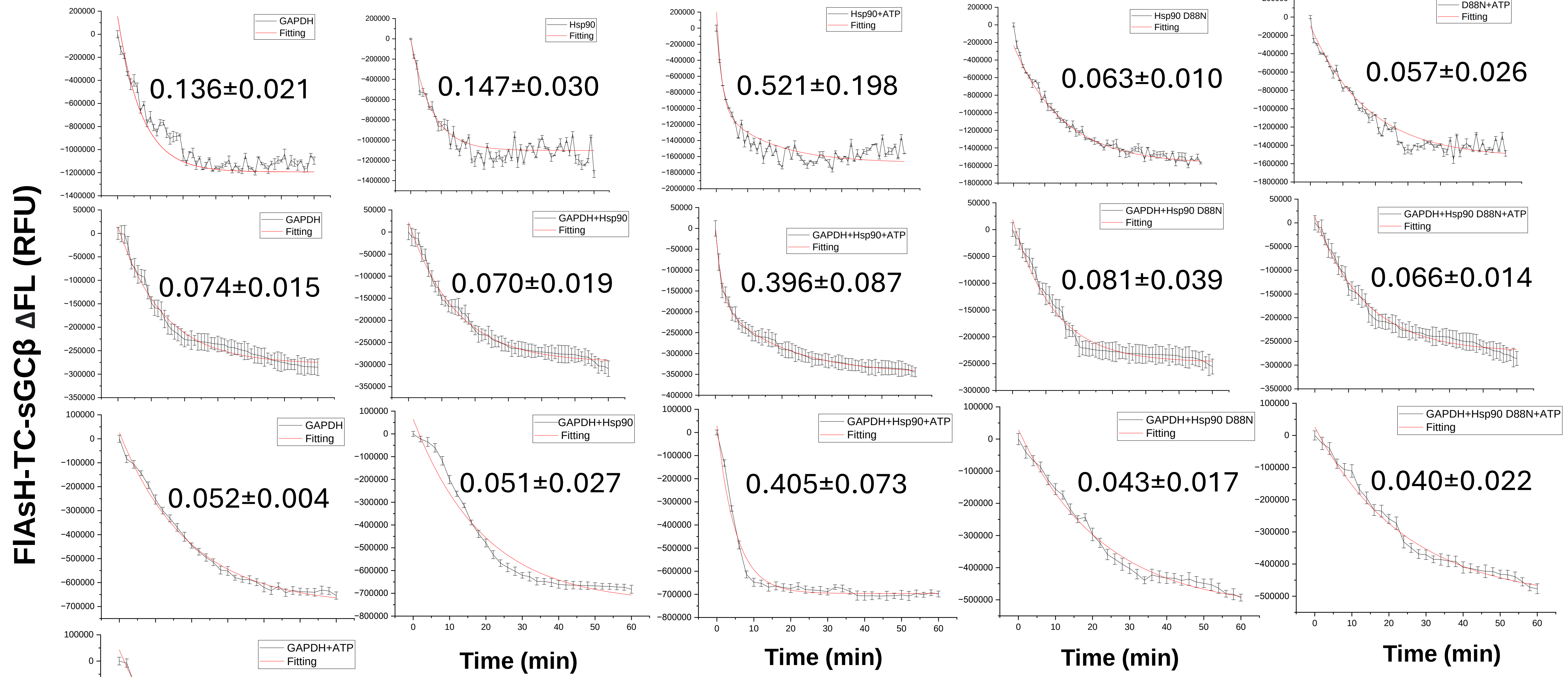

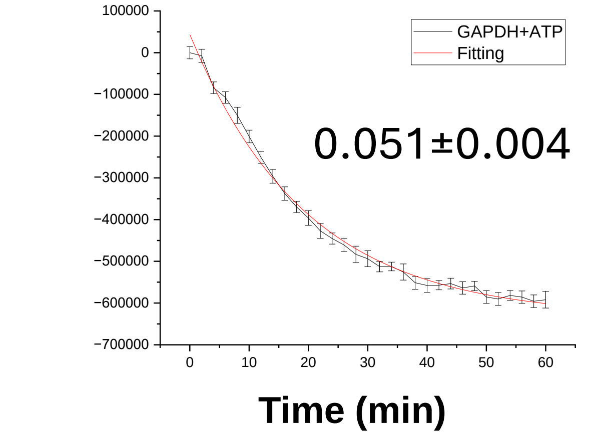

Fig. S6. The impact of Hsp90 and its ATPase activity on GAPDH heme transfer into FlAsH-TC-apo-sGCβ. Reactions contained 1 µM FlAsH-TC-apo-sGCβ alone or in complex with Hsp90. Reactions were initiated by adding the GAPDH-heme complex and the FlAsH fluorescence emission was recorded vs time. In some cases, ATP was present or variants of Hsp90 (D88N) or TC-apo-sGCβ (HD) were used as indicated. Panels show the mean fluorescence traces (+/- SD of three replicates) and the lines of best fit (single exponential, red) and report the mean rate derived for each kinetic trace (min^{-1}) which were also used to create the main Fig. 3C. For each reaction condition, three independent trials were run (3 panels each) except for the reaction condition where ATP was added alone (1 trial and 1 panel).

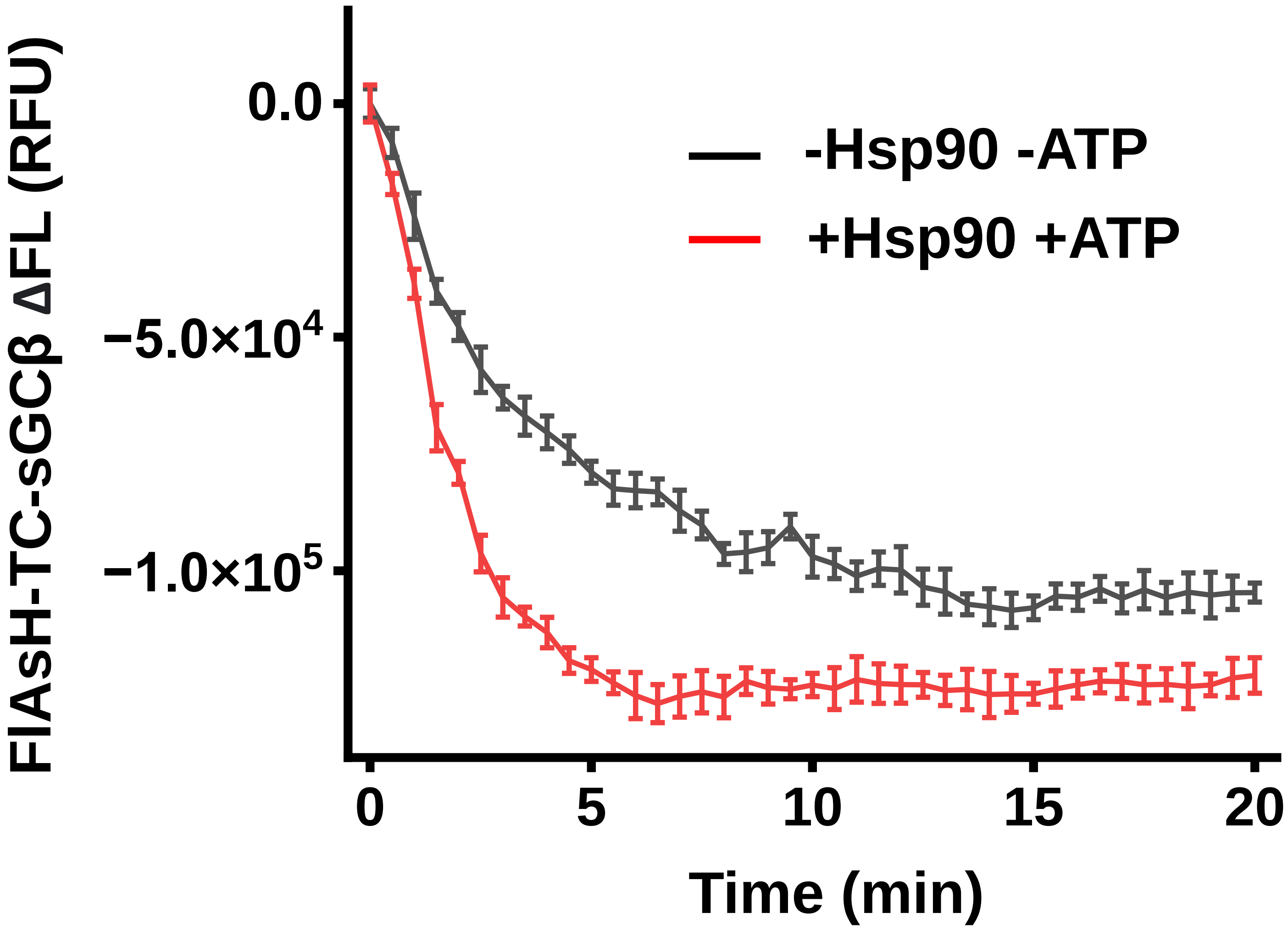

Fig. S7. Hsp90/ATP stimulation of heme transfer into the FlAsH-TC-apo-sGCβ is independent of the added GAPDH-heme complex concentration. Reactions contained 1 μM FlAsH-TC-apo-sGCβ alone or in complex with Hsp90 (+/- ATP) and were initiated by adding the GAPDH-heme complex at a 3.75-fold molar excess. The FlAsH fluorescence emission was recorded every 30 s for 20 min. The panel shows the mean fluorescence traces (+/- SD of three replicates) and is representative of two independent trials.

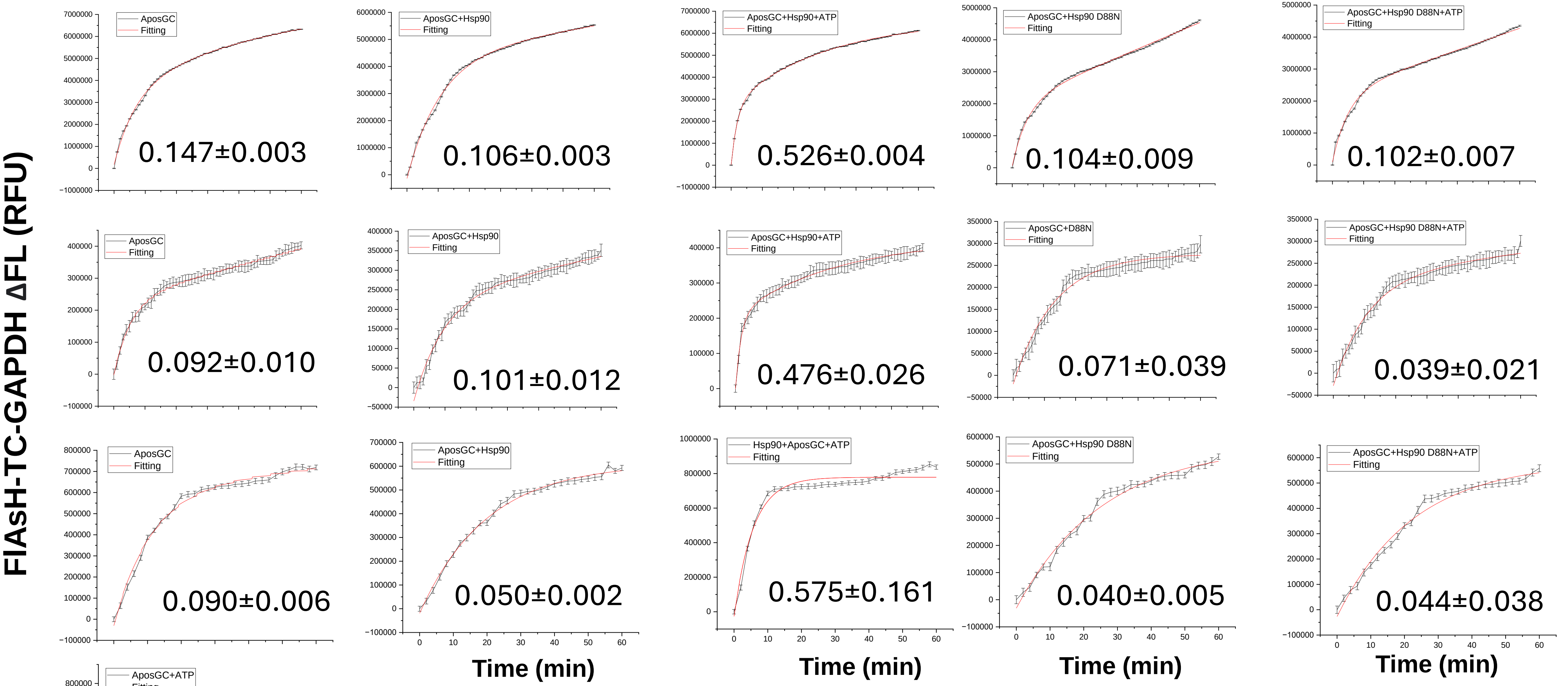

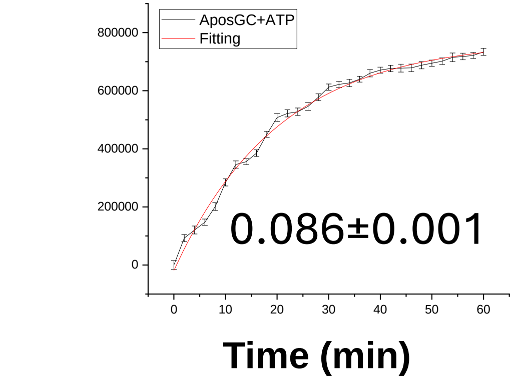

Fig. S8. The impact of Hsp90 and its ATPase activity on heme transfer from FlAsH-TC-GAPDH to apo-sGCβ. Reactions contained 1 μM FlAsH-TC-GAPDH heme complex and were initiated by adding apo-sGCβ alone or in complex with Hsp90. The FlAsH fluorescence emission was recorded vs time. In some cases, ATP was present or variants of Hsp90 (D88N) were used as indicated. Panels show the mean fluorescence trace +/- SD of three replicates and the lines of best fit (double exponential, red) and report the initial rate (k_1, min^{-1}) derived for each kinetic trace, which were also used to create the main Fig. 3D. For each reaction condition, three independent trials were run (3 panels each) except for the reaction condition where ATP was added alone (1 trial and 1 panel).

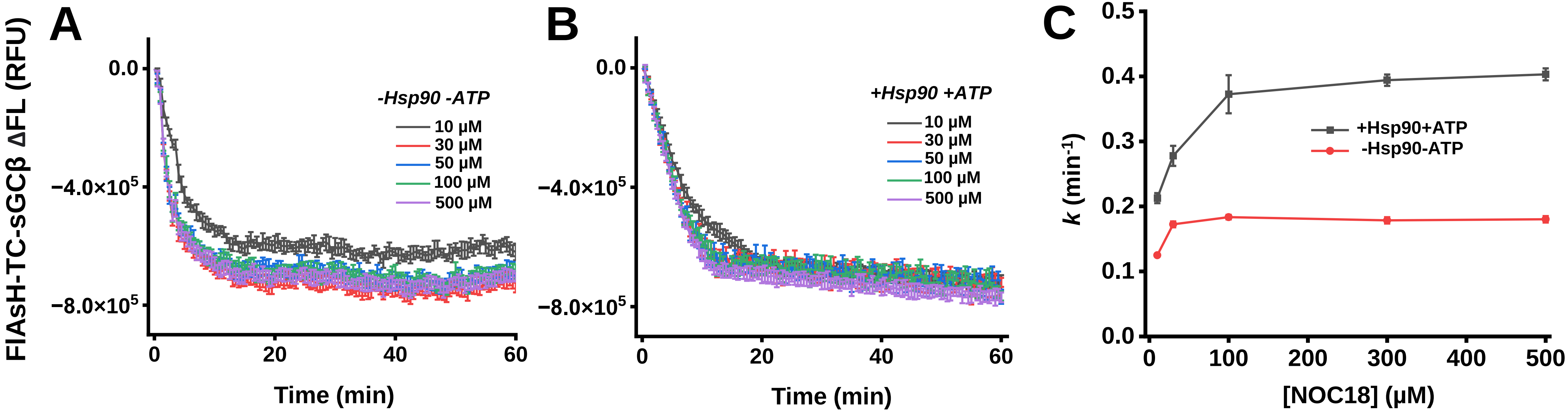

Fig. S9. NO increases the rate of heme transfer from GAPDH into FlAsH-TC-apo-sGCβ. Reactions contained 1 μM each of GAPDH-heme complex and FlAsH-TC-apo-sGCβ either (*A*) alone or (*B*) in complex with Hsp90 (plus ATP) and were initiated by adding the GAPDH-heme complex, with NOC18 being present in the reactions at the indicated concentrations. The fluorescence traces shown in *A and B* are the mean +/- SD of 3 replicates and are representative of two independent trials. The rates derived from fitting the traces to a single exponential equation are plotted in Panel *C*.

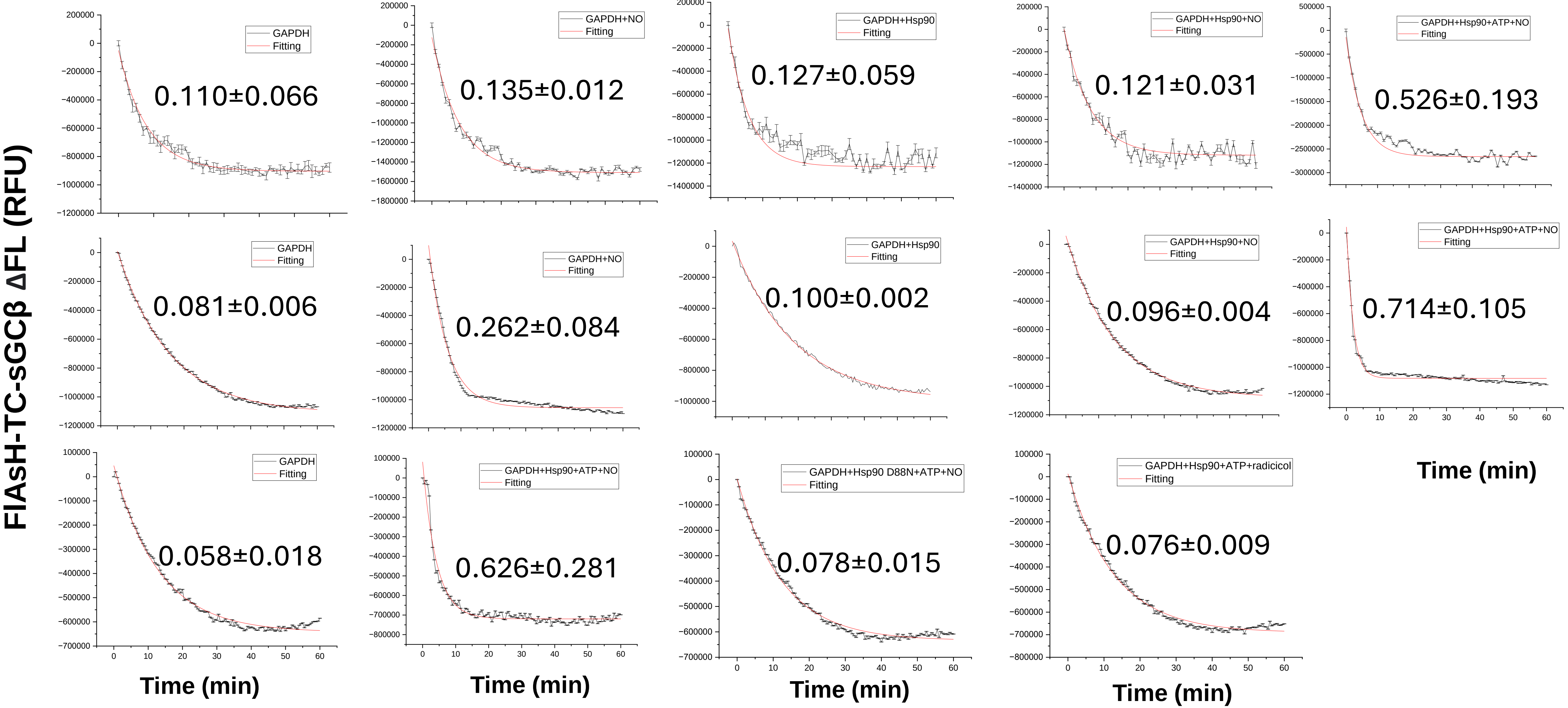

Fig. S10. Effect of NO on GAPDH heme transfer into FlAsH-TC-apo-sGCβ. Reactions contained 1 µM FlAsH-labeled TC-apo-sGCβ alone or in complex with Hsp90 and were initiated by adding the GAPDH-heme complex to give a final concentration of 1 µM. In some reactions NOC18, ATP, or and/or radicicol were also present or a variant of Hsp90 (D88N) was used as indicated. Panels show the mean fluorescence traces (+/- SD of three replicates) and the lines of best fit (single exponential, red) and report the rate derived for each kinetic trace (min^{-1}) which were also used to create the main Fig. 4C. For each reaction condition, three independent trials were run (3 panels each) except for the reactions where D88N Hsp90 or radicicol were used (1 trial and 1 panel each).

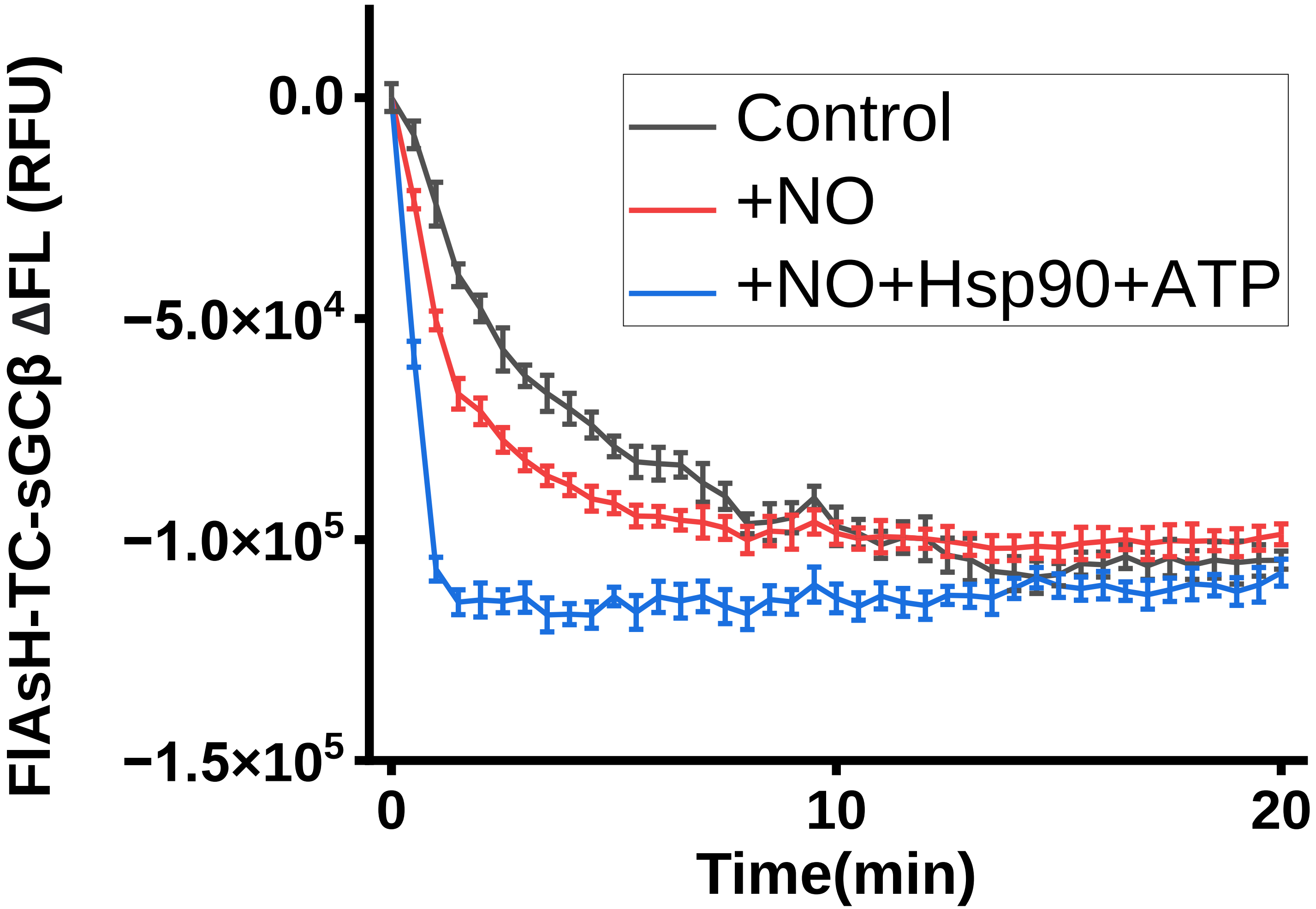

Fig S11. The enhancing effect of NO on heme transfer into FlAsH-TC-apo-sGCβ is independent of the amount of GAPDH-heme complex in the reaction. Reactions contained 1 µM FlAsH-TC-apo-sGCβ alone or in complex with Hsp90 (plus ATP) and were initiated by adding the GAPDH-heme complex at a 3.75-fold molar excess. NOC18 was present as indicated. The FlAsH fluorescence emission was recorded every min for 20 min. The panel shows the mean fluorescence traces +/- SD of three replicates for each reaction condition and is representative of two independent trials.

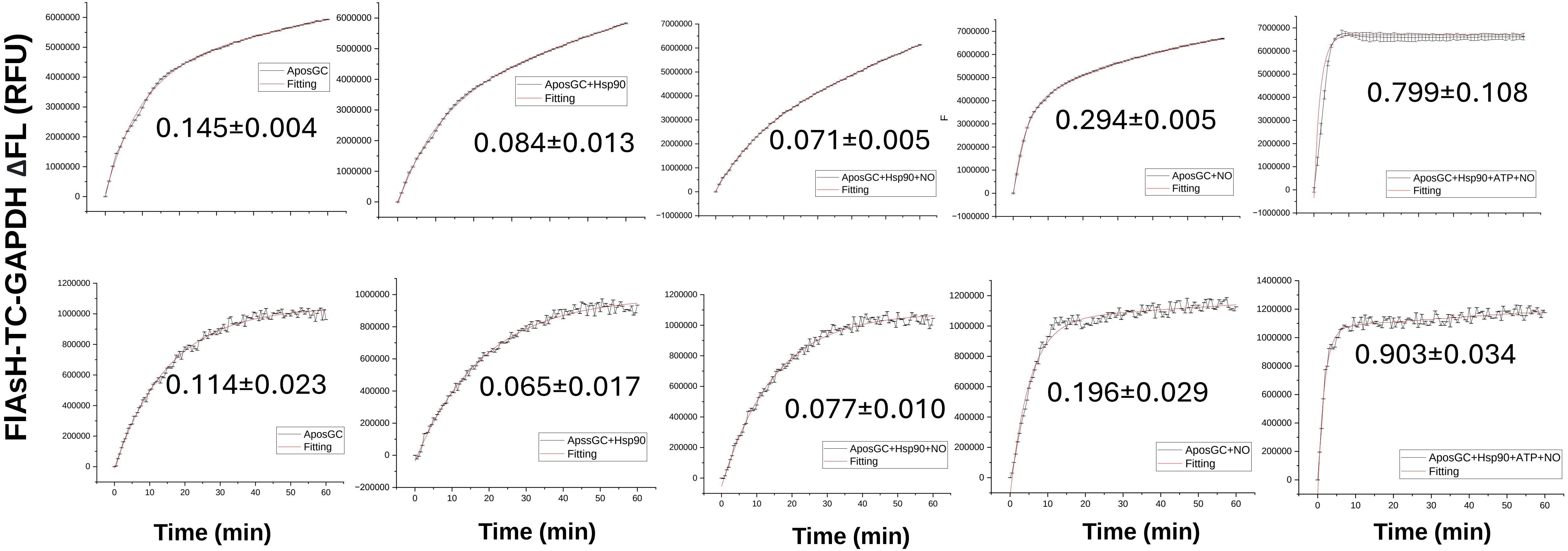

Fig. S12. Effect of NO on heme transfer from FlAsH-TC-GAPDH to apo-sGCβ. Reactions contained 1 μM of FlAsH-TC-GAPDH-heme complex and were initiated by adding apo-sGCβ either alone or in complex with Hsp90. In some reactions NOC18 and/or ATP was present as indicated. Panels show the mean fluorescence traces +/- SD of three replicates and the lines of best fit (single exponential, red) and report the rate derived for each kinetic trace (min^{-1}) which were also used to create the main Fig. 5B. For each reaction condition, trials were done three independent times (3 panels each) except for the condition where the reaction contained Hsp90, ATP, and NOC18 (1 trial and 1 panel each).

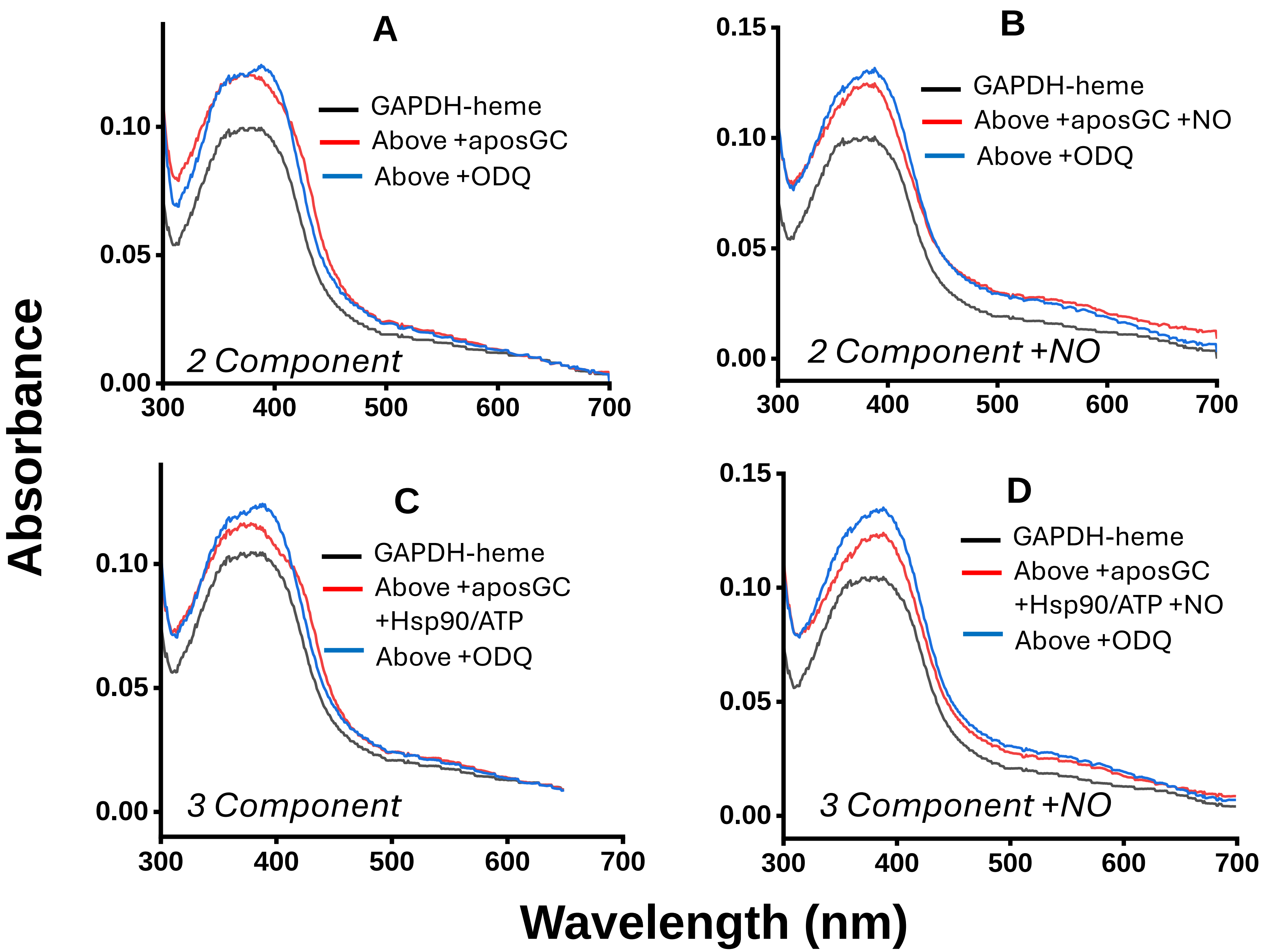

Fig. S13. UV-visible spectra recorded before and after initiating a heme transfer reaction between GAPDH heme complex and apo-sGCβ. Reactions contained 1 µM GAPDH-ferric heme complex and were initiated by adding apo-sGCβ alone (Panels *A and B*) or in complex with Hsp90 (Panels *C and D*) to give 1 µM final concentration, and then were run for 30 min. Some reactions contained 100 µM NOC18 as indicated (Panels *B and D*). All reactions were given ODQ after 30 min and the incubated a further 30 min. Traces shown are the spectra recorded at reaction time = 0 (black), at 30 min after initiating the reaction (red), and at 30 min after adding ODQ (blue). Traces are representative of three independent trials.

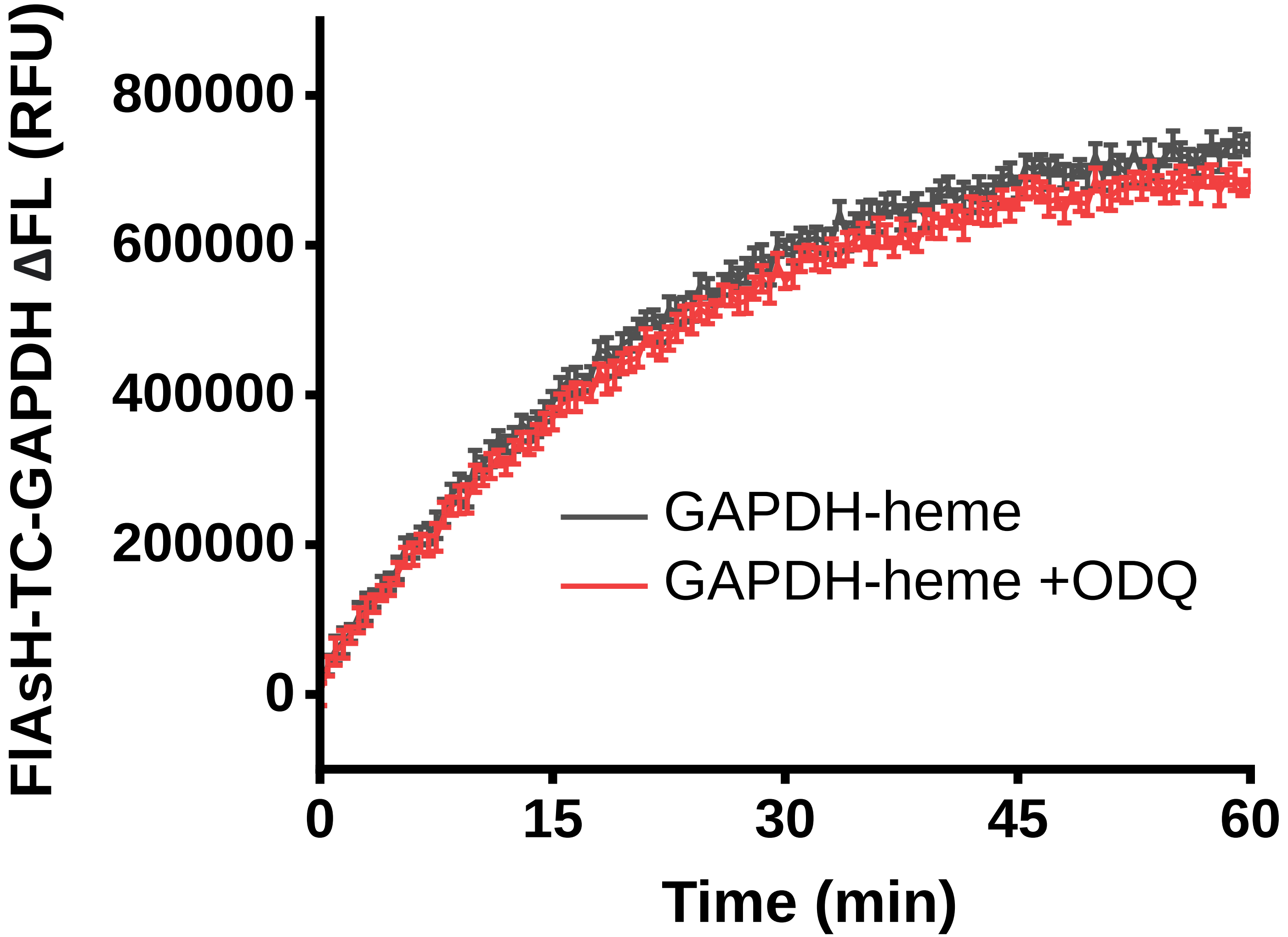

Fig. S14. ODQ does not alter the rate of ferric heme dissociation from GAPDH. Reactions contained a FlAsH-TC-GAPDH ferric heme complex in presence or absence of 10 μM ODQ and were initiated by adding a 30-fold molar excess of GAPDH. The fluorescence traces shown are representative of two or three independent trials.

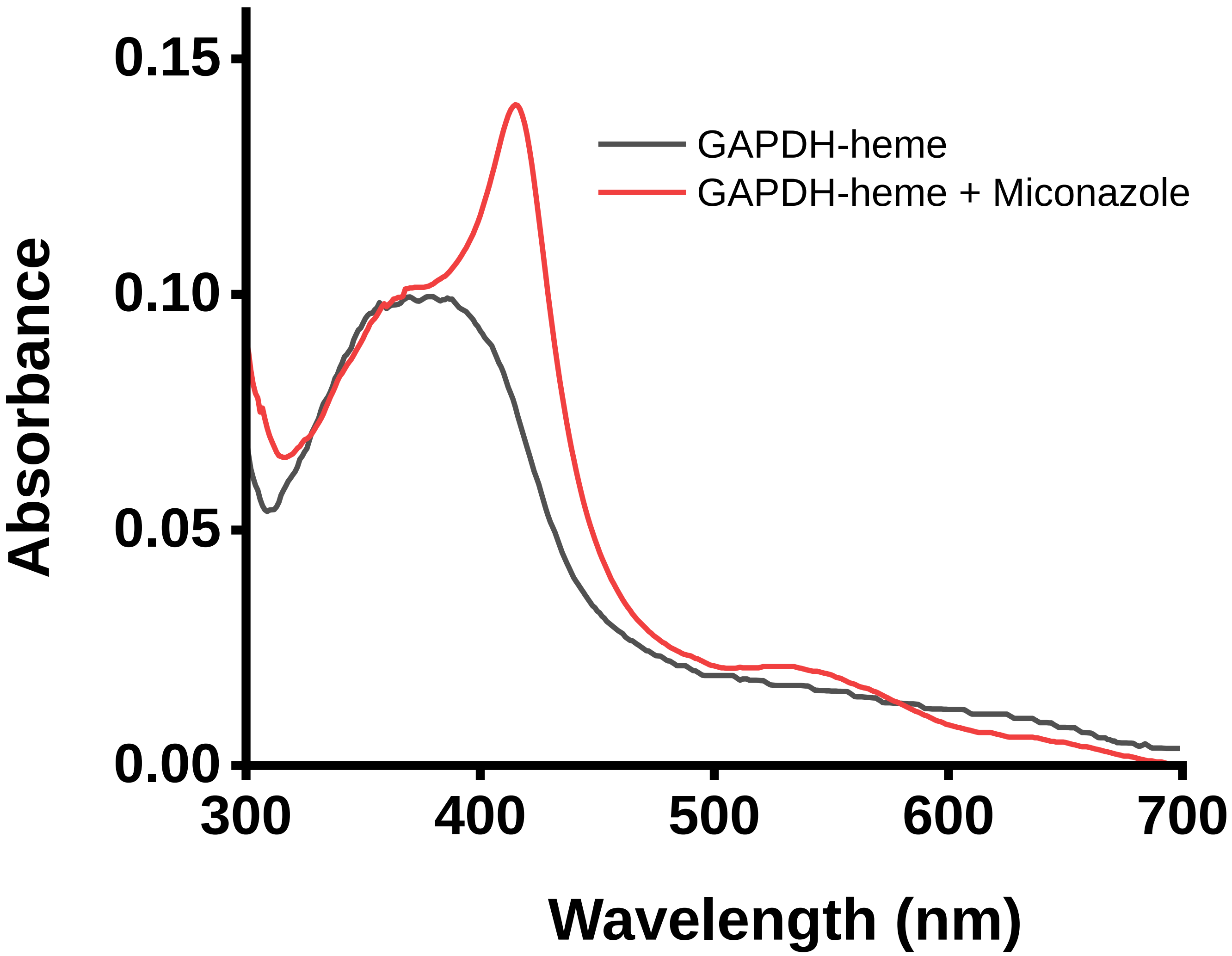

Fig. S15. UV-visible spectra of Miconazole-bound GAPDH-heme complex. 1 μM GAPDH-heme complex alone (black) or in the presence of 5 μM of miconazole (red). Traces are representative of three independent trials.

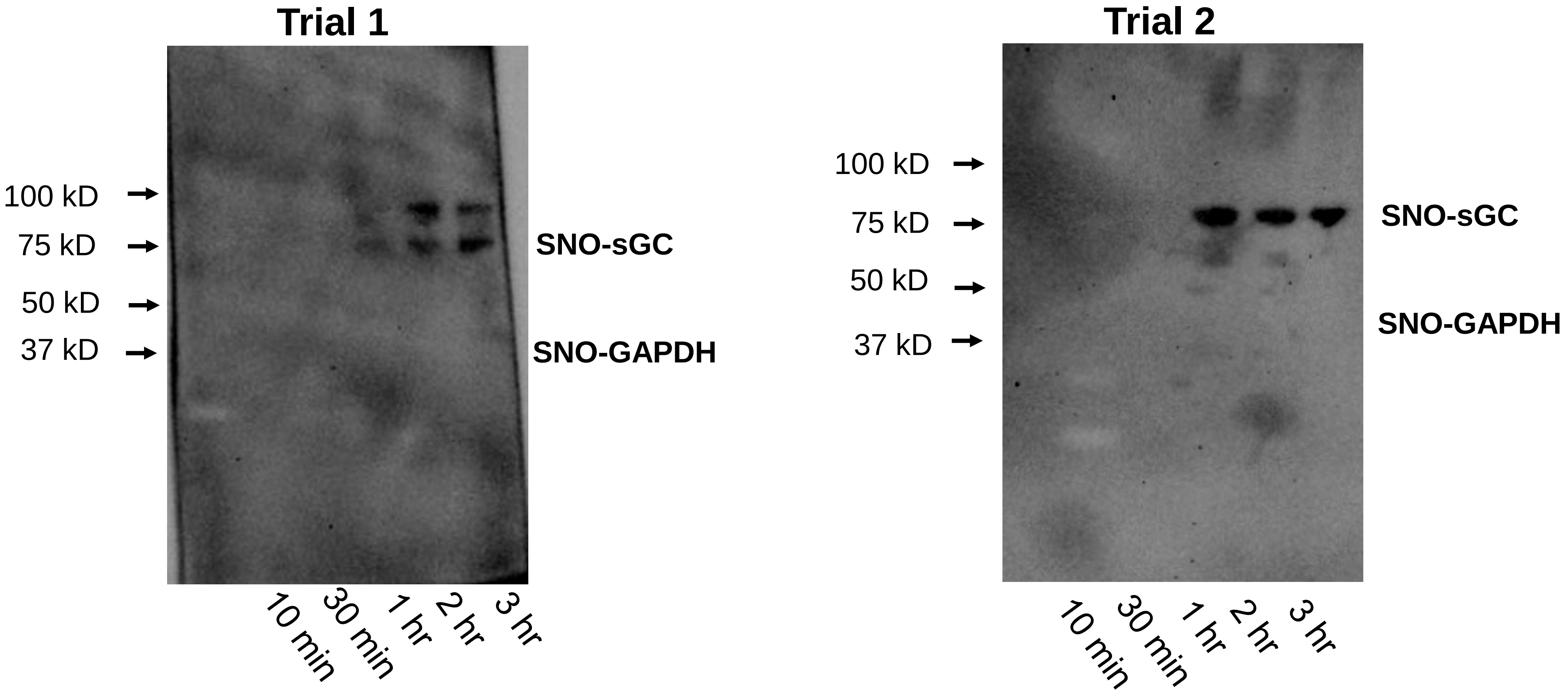

Fig. S16. Cys-NO (SNO) buildup in apo-sGCβ or GAPDH after addition of NOC18. GAPDH-heme complex was mixed with apo-sGCβ in complex with Hsp90 plus ATP to give final protein concentrations of 1 µM each and then had 100 µM NOC18 added at time = 0. Reaction samples were removed at the indicated time points for protein SNO analysis. SNO modification in either protein was detected by Western blot using the biotin switch method. Representative Western blots are shown, with the band intensities indicating the relative level of SNO modification in each protein versus time. Results of two independent trials were shown.

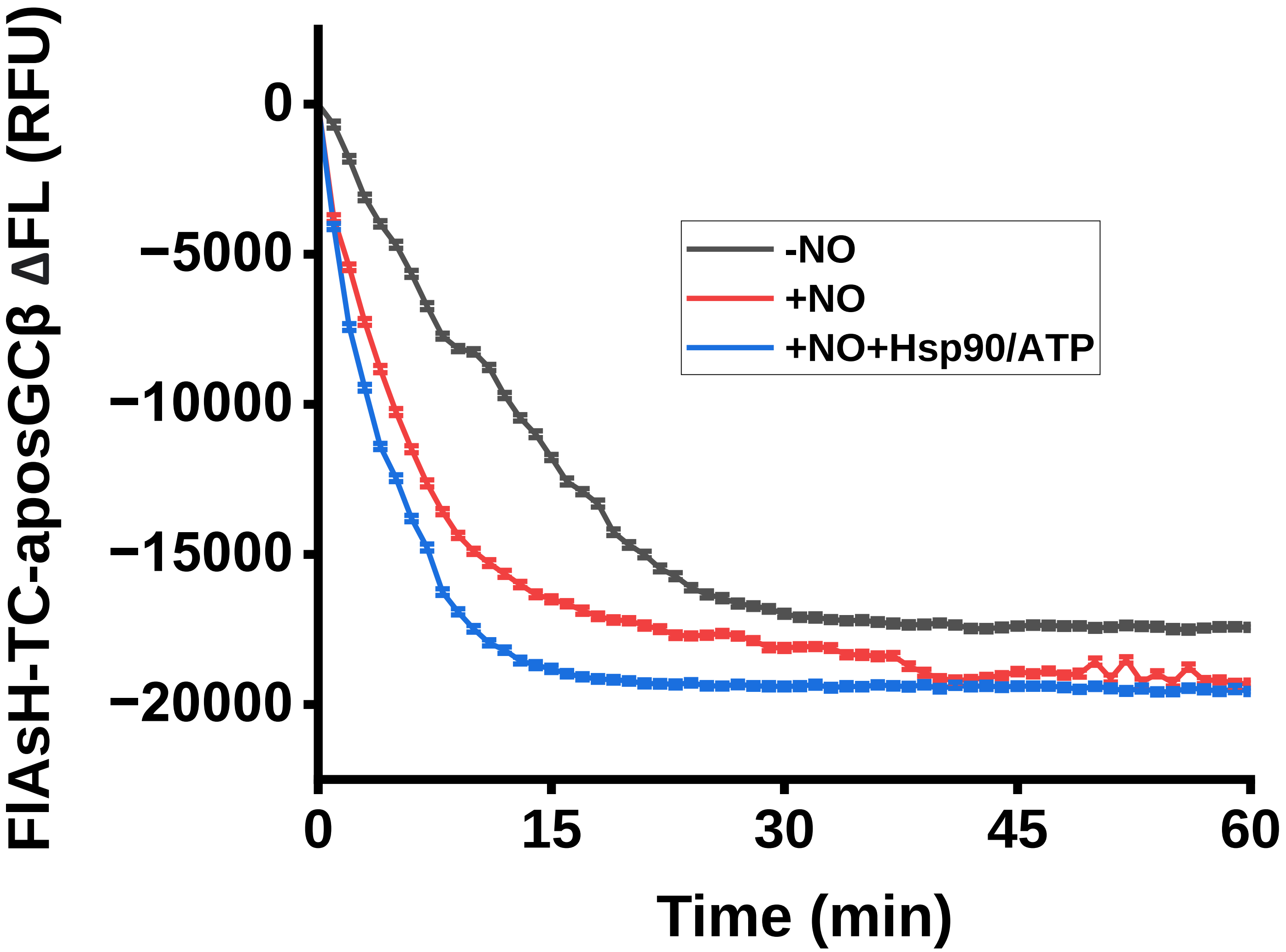

Fig. S17. Conversion of three Cys residues in GAPDH to Ser does not alter its ability to transfer heme to FlAsH-TC-apo-sGCβ. Reactions contained 1 µM of FlAsH-TC-apo-sGCβ either alone or in complex with Hsp90 (plus ATP) and were initiated by adding the GAPDH (3-Cys to Ser) variant containing ferric heme. NOC18 was added as indicated. The traces show the mean fluorescence readings for 3 replicates taken every min for 60 min. Traces are representative of two independent trials.
